# Quantitative diffusion measurements using the open-source software PyFRAP

**DOI:** 10.1038/s41467-018-03975-6

**Published:** 2018-04-20

**Authors:** Alexander Bläßle, Gary Soh, Theresa Braun, David Mörsdorf, Hannes Preiß, Ben M. Jordan, Patrick Müller

**Affiliations:** 1 Friedrich Miescher Laboratory of the Max Planck Society, Max-Planck-Ring 9, 72076 Tübingen, Germany; 2 Department of Organismic and Evolutionary Biology, Harvard University, 26 Oxford Street, Cambridge, MA 02138 USA; 3 Present Address: University of Konstanz, Universitätsstraße 10, 78457 Konstanz, Germany

## Abstract

Fluorescence Recovery After Photobleaching (FRAP) and inverse FRAP (iFRAP) assays can be used to assess the mobility of fluorescent molecules. These assays measure diffusion by monitoring the return of fluorescence in bleached regions (FRAP), or the dissipation of fluorescence from photoconverted regions (iFRAP). However, current FRAP/iFRAP analysis methods suffer from simplified assumptions about sample geometry, bleaching/photoconversion inhomogeneities, and the underlying reaction-diffusion kinetics. To address these shortcomings, we developed the software PyFRAP, which fits numerical simulations of three-dimensional models to FRAP/iFRAP data and accounts for bleaching/photoconversion inhomogeneities. Using PyFRAP we determined the diffusivities of fluorescent molecules spanning two orders of magnitude in molecular weight. We measured the tortuous effects that cell-like obstacles exert on effective diffusivity and show that reaction kinetics can be accounted for by model selection. These applications demonstrate the utility of PyFRAP, which can be widely adapted as a new extensible standard for FRAP analysis.

## Abstract (web summary)

FRAP analysis often relies on simplified assumptions that can affect measurement accuracy. Here the authors present a Python-based FRAP analysis software using simulations instead of simplified theoretical models to fit the data, which accounts for complex sample geometries and bleach conditions.

## Introduction

**Fig. 1:**
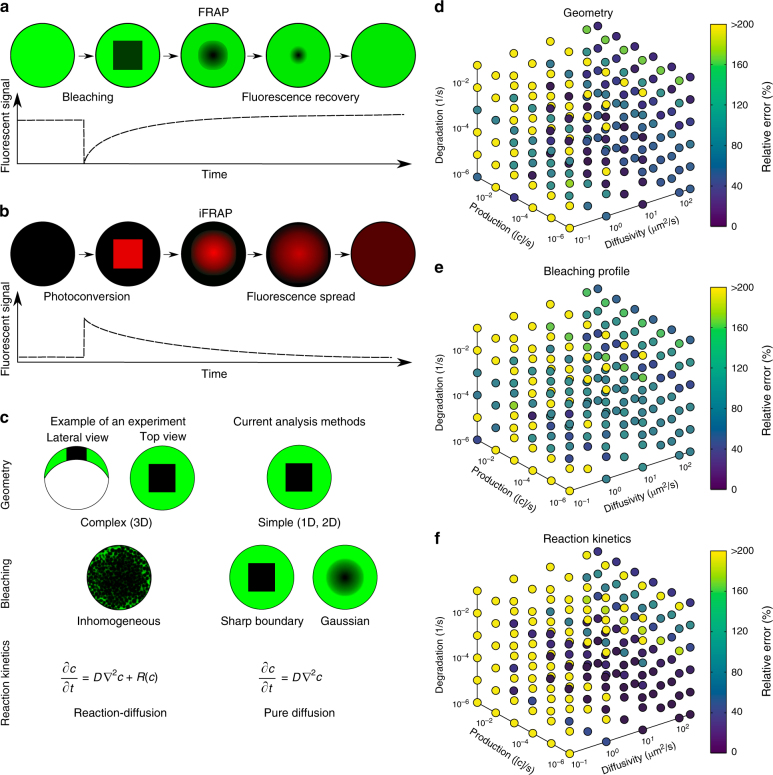
Fluorescence perturbation techniques used for effective diffusion measurements, and drawbacks of current analysis methods. **a** In fluorecence recovery after photobleaching (FRAP) experiments, a small region in the sample is bleached. After bleaching, the diffusion-driven recovery in the bleached region is monitored. **b** Inverse FRAP (iFRAP) is an experimental mirror image of FRAP: Molecules in a given region are photoconverted and then spread throughout the sample, resulting in the loss of fluorescent signal in the region of photoconversion. **c** Drawbacks of current analysis methods exemplified with zebrafish development at late blastula stages. Current analysis methods simplify sample geometry, idealise bleaching profiles, or ignore underlying reaction kinetics. **d**–**f** Possible relative error in diffusion coefficient estimates that can occur if false assumptions are made about sample geometry (**d**), bleaching conditions (**e**), or reaction kinetics (**f**), respectively. The maximum displayed error was capped to a value of 200%, but can be up to 1000%

The diffusion of molecules is important for almost any process across all scales of biological organisation, from transcription factors finding their targets on DNA to signalling molecules spreading through tissues during development and homoeostasis^1–3^. The biological function of a molecule is affected by its action range and therefore its mobility; however, effective diffusion of molecules moving through complex tissues is difficult to measure quantitatively. More than 40 years ago, Poo & Cone^4^ and Liebman & Entine^5^ developed a method to assess the diffusivities of fluorescent molecules. In these fluorescence recovery after photobleaching (FRAP) experiments, the fluorescence of molecules in a small region of the sample is bleached by exposure to a strong laser pulse^6^. The dynamics of fluorescence recovery in the bleached region can then be used to infer the mobility of the fluorescent molecules (Fig. 1a). Inverted FRAP (iFRAP) assays have recently been developed as an extension of FRAP experiments^7–10^, which eliminate the often harsh bleaching conditions used in FRAP experiments. iFRAP assays utilise photoconvertible molecules that can be induced to alter their fluorescence excitation/emission properties after exposure to ‘photoconverting’ light. In iFRAP experiments, the spread of signal from a small photoconverted domain into the neighbouring regions of the sample is monitored over time and thus represents an experimental mirror image of FRAP (Fig. 1b).

Diffusion coefficients are commonly extracted from FRAP experiments by fitting analytical solutions computed from theoretical models to the measured recovery curves^11–18^, and a few simulation-based analysis methods have been developed^19–21^. Although this allows for a rapid assessment of qualitative mobility differences in identical experimental settings, current approaches rely on several assumptions that can affect the accuracy of the analysis. First, most current methods reduce the FRAP analysis to one-dimensional or two-dimensional simplifications^11–21^, often assuming that the fluorescent pool is infinitely large^11–14,16,17^, or ignoring more complex geometries of biological samples that could play important roles in molecule movement (Fig. 1c). Recent studies have argued that geometry is crucial for dynamic biological processes^22,23^, and must be taken into account for accurate analysis of FRAP data. Indeed, false assumptions about the FRAP sample geometry can drastically affect diffusion coefficient estimates (Fig. 1d).

Second, the bleaching process in FRAP experiments is often inaccurately modelled. Bleaching is posited to be homogeneous or to follow a Gaussian distribution throughout bleached circular or rectangular regions, while the molecules outside of the bleached region are assumed to remain unbleached^11–13, 15–18^. However, molecules diffusing during the bleaching process can create inhomogeneities both inside and outside of the bleached region; moreover, a delay between bleaching and the start of the recovery measurement can lead to further inhomogeneities (Fig. 1c). Incorrect assumptions about the bleaching process can thus lead to a severe misestimation of diffusion coefficients^14, 24–27^ (Fig. 1e).

Third, in vivo FRAP experiments can be strongly influenced by reaction kinetics such as production or degradation of fluorescent molecules, which can contribute to the observed recovery curve (Fig. 1c). However, this is mostly neglected in classical FRAP analysis models and can lead to erroneous diffusion estimates (Fig. 1f)^11–17^.

**Fig. 2:**
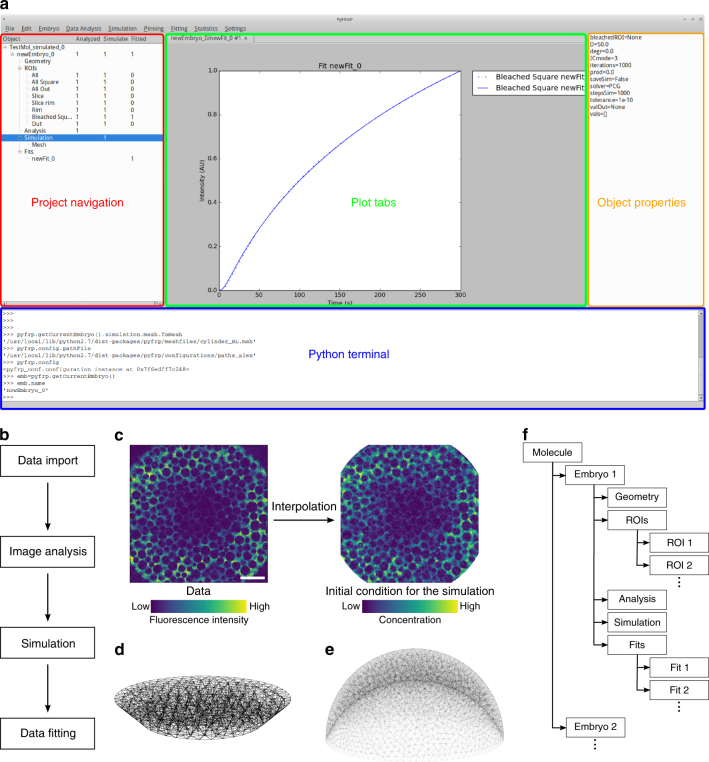
The PyFRAP software package. **a** Annotated snapshot of the PyFRAP main GUI with project navigation tree (red), plot tabs (green), object property display (orange), and integrated Python terminal (blue). **b** PyFRAP work flow. **c** PyFRAP’s interpolation of the first post-bleach image as initial condition for FRAP simulations. The length of the white scale bar represents 100 μm. **d**,** e** Spatial discretisation of geometries resembling **d** a frustum and **e** a zebrafish embryo at late blastula stages (dome stage). **f** PyFRAP’s data structure

To address these shortcomings, we developed the versatile Python-based FRAP analysis software PyFRAP (available at https://mueller-lab.github.io/PyFRAP). To facilitate data analysis, PyFRAP is equipped with an intuitive graphical user interface (GUI, Fig. 2a), which gives users without a computational background access to a sophisticated FRAP data analysis work flow from image analysis to statistical model comparison methods (Fig. 2b). PyFRAP applies the first post-bleach image as initial condition (Fig. 2c), and numerically simulates the FRAP experiment in realistic two-dimensional or three-dimensional experiment geometries (Fig. 2d, e); the solution from this simulation is then fitted to the experimental data. Furthermore, PyFRAP can accurately account for both uniform production and degradation during FRAP experiments. PyFRAP saves all analysed data and settings in a logical data structure that can be shared with collaborators or re-used for later analyses (Fig. 2f). The software is freely available, and the open-source environment allows for rapid expansion through collaborative work^28^ to adjust analysis methods to the users’ needs.

To demonstrate the utility of PyFRAP, we conducted several typical in vitro and in vivo FRAP experiments (Supplementary Fig. 1). PyFRAP accurately determines the diffusion coefficients of fluorescent molecules ranging from 3 to 500 kDa in both artificial and biological contexts. In contrast to currently available software, PyFRAP’s flexible initial conditions also allow analysis of iFRAP experiments, producing results comparable to FRAP. We used PyFRAP to measure the influence that obstacles such as cells exert on the movement of diffusing molecules, and found that such geometric hindrance decreases diffusivity by about one-third. Moreover, PyFRAP provides accurate modelling of reaction kinetics, including production and degradation. Finally, to test the impact of extracellular binding on protein diffusivity, we measured the diffusion of signalling molecules in living zebrafish embryos. We found that the effective diffusivity of a signalling molecule in developing zebrafish was reduced to about one-tenth of its predicted value, in agreement with hindered diffusion models postulating interactions of embryonic signals with diffusion regulators^22,29^. Altogether, our analyses highlight how detailed examination of FRAP data can be used to determine the contribution of individual factors to the movement of molecules in controlled artificial and biological contexts^30^.

## Results

### PyFRAP is a versatile FRAP/iFRAP analysis package

Current FRAP analysis methods often make simplified assumptions about FRAP experimental conditions to aid in the derivation of analytical solutions^11–16,18^, and to facilitate numerical simulations^20,21^. Such assumptions include reducing complex sample geometries to lower dimensions, idealising the initial bleaching profile, or ignoring additional reaction kinetics potentially underlying fluorescence recovery (Fig. 1c). Unless the experiment is well approximated by these assumptions (e.g., simple geometry, small bleach spot compared to a large sample volume, sharp bleach profile, no reactions), this can lead to erroneous diffusion estimates (Fig. 1d–f). To address these shortcomings, we developed PyFRAP. PyFRAP numerically simulates FRAP experiments in realistic three-dimensional geometries using an interpolation of the first post-bleach image as initial condition. This simulation is then fitted to the experimental data, incorporating reaction kinetics such as uniform production and degradation.

PyFRAP is an open-source Python-based FRAP analysis software that runs on the major operating systems Microsoft Windows, Mac OSX and Linux. Over the past 20 years, Python has become the standard programming language for scientific research because of the availability of versatile add-on packages and its intuitive and simple syntax^31^. Building on the resourcefulness of Python, PyFRAP is based on commonly used packages such as PyQT, SciPy and FiPy^32–36^. PyFRAP comes with an intuitive graphical user interface (GUI, Fig. 2a) and a fully documented application programming interface (API) allowing quick development of scripts or modifications of the PyFRAP code. PyFRAP’s functionalities include sophisticated image processing functions useful for FRAP analysis, customisable geometry and analysis region definitions, a finite element partial differential equation (PDE) solver that simulates FRAP/iFRAP experiments with adjustable options, statistical tools for averaging and model comparison, and multiple plotting and input/output functions (see Methods section and Supplementary Note 1 for details). To make the software easily accessible, dialogue boxes (software wizards) guide the user step-by-step through data import, image analysis, simulation and fitting.

We programmed PyFRAP to import image data from most common microscope formats, such as .tif, .lsm and .czi. Users can define arbitrary regions of interests (ROIs) that are then used for image analysis, simulation and fitting (Supplementary Fig. 2a). For some experimental setups, the imaged sample might be larger than the field of view. In these cases, the concentration of molecules in regions outside of the image can be estimated from selected areas in the first image of the recovery image series (Supplementary Fig. 2b). Uneven illumination is a common artefact in FRAP experiments. PyFRAP can correct this artefact by normalisation using pre-bleach images or using a correction matrix computed from a secondary data set generated with a homogeneously distributed fluorophore^37–39^ (see Methods section and Supplementary Fig. 2c for details). To avoid numerical instabilities, PyFRAP allows the user to smooth or denoise the image data using a Gaussian or median filter (see Methods section, Supplementary Note 1, Supplementary Fig. 3, and Supplementary Table 1 for details).

FRAP and iFRAP experiments have been performed in a variety of contexts, from the cigar-shaped *Drosophila* embryo and the relatively flat *Drosophila* wing disc to the dome-shaped pre-gastrula stage zebrafish embryo^10,22,29, 40–42^. These structures have distinct geometries that could impact fluorescence recovery. In fact, we found that simplifying the three-dimensional zebrafish embryo to a two-dimensional disc can frequently lead up to a >200% error in estimated diffusion coefficients (Fig. 1d). In PyFRAP, users can define arbitrary two-dimensional and three-dimensional geometries using Gmsh^43^ or CAD STereoLithography (.stl) files that are then spatially discretised into tetrahedral meshes by Gmsh in combination with TetGen^44^. PyFRAP provides various meshing options, such as local mesh refinements, boundary layer meshes and attractor meshes, allowing users to adapt the mesh to experimental details (see Fig. 2d, e and Supplementary Fig. 4c for example geometries and meshes).

In current FRAP analysis methods, the initial condition of the FRAP experiment is often simplified to a simple rectangular function or a Gaussian profile to approximate sharp or blurred bleach boundaries, respectively^11,12, 14–18, 45–47^. However, light scattering, imperfect bleaching and diffusion during the bleaching process can lead to more complex bleaching profiles and thus need to be considered during FRAP analysis to avoid misestimation of diffusion coefficients^24,25,30,48^. To overcome this issue, PyFRAP uses a bilinear interpolation between pixels of the first post-bleach image to estimate the initial condition for mesh cells. This initial condition closely resembles initial experimental bleaching profiles and concentration distributions (Fig. 2c). Moreover, in contrast to most current FRAP analysis methods^11–18,46,47^, PyFRAP does not fit a mathematical expression based on simplified assumptions to the data; instead, PyFRAP uses FiPy^32^ to simulate the experiment numerically, resulting in a solution that incorporates the realistic three-dimensional geometry and initial conditions. The numerical simulation is then fitted to the FRAP data by minimising the sum of squared differences using classical optimisation algorithms^49–51^ (see Methods section for details).

In typical FRAP and iFRAP experiments, a protein of interest is tagged with a fluorescent protein and expressed within a tissue. In such an experiment, the fusion protein is often actively produced at the same time that FRAP is carried out; additionally, fusion proteins undergo degradation over time. Depending on how the fusion protein is expressed (promoter-driven expression, mRNA injection, etc.), its degradation kinetics, and the timescale of the FRAP/iFRAP experiment, production and degradation can dramatically influence recovery curves. Ignoring reaction kinetics in FRAP experiments could therefore lead to erroneous diffusion coefficient estimates. Indeed, recovery curves with pure diffusion fitted to a simulated reaction-dominant data set often resulted in a >200% error in the estimated diffusion coefficients (Fig. 1f). To ensure that the appropriate reaction kinetics are considered when analysing FRAP data, PyFRAP is equipped with four models: (1) Pure diffusion, (2) diffusion with production, (3) diffusion with degradation and (4) diffusion with production and degradation (see Methods section for details). The model can be constrained with previous reaction rate measurements from assays such as fluorescence decay after photoconversion (FDAP)^52,53^; alternatively, production and degradation rates can be directly obtained from fitting the FRAP data. Below, we discuss methods to determine which approaches are most appropriate for a given data set.

An advantage of PyFRAP is its ability to assess FRAP data using multiple models of varying complexity, from pure diffusion to combined reaction-diffusion kinetics. However, determining which model is appropriate for a given data set can be challenging. Choosing the incorrect model can lead to overfitting and potentially false diffusion coefficients^54^. The Akaike information criterion (AIC) is a statistical tool that can aid in model selection^55^. PyFRAP’s implementation of the AIC allows users to compare the models mentioned above and determines the most likely model based on a relative weighted measure that includes both the model’s log-likelihood and its degrees of freedom, i.e., the number of model parameters. Moreover, PyFRAP provides several statistical tests (Supplementary Table 2) to assess differences between measurements and obtained fits, such as Student’s *t*-test^56^ for normally distributed data or the Mann–Whitney-U-test^57^, which does not require normally distributed data. The Shapiro–Wilk-test can be used to assess whether the measured diffusivities follow a normal distribution^58^ and whether application of Student’s *t*-test or the Mann–Whitney-U-test is justified.

PyFRAP’s object-oriented data structure (Fig. 2f) can be saved into serialised objects and easily loaded for further analysis or shared with collaborators. In addition, PyFRAP lets users visualise every aspect of PyFRAP’s analysis work flow and save plots and images into publication-ready figures.

### Benchmarking PyFRAP

To validate PyFRAP, we first determined whether it can recover true diffusion coefficients and reaction kinetics from simulated data. We used our previous in-house solution^22,29,42^ based on the commercial programs MATLAB and COMSOL multiphysics to simulate 24 FRAP experiments with different reaction kinetics and diffusion coefficients. Using PyFRAP, the simulated data sets were fitted with all four possible reaction-diffusion models (see above). We determined a maximal error of 10% (average error: 2%, Supplementary Table 3) between simulated and estimated diffusion coefficients, demonstrating that PyFRAP recovers correct diffusion coefficients within the error tolerance of the numerical simulations.

Next, we tested whether PyFRAP’s implementation of the AIC allows identification of the models used to create the simulated data. When the data were simulated with models describing either pure diffusion, diffusion and degradation, or diffusion and production, the AIC predicted the correct underlying model (Supplementary Table 3). However, the model selection based on the AIC did not favour the correct model for data sets that included diffusion combined with both production and degradation, since models with fewer degrees of freedom provided smaller Akaike weight values. Simulations involving diffusion, production and degradation can generate data effectively indistinguishable from data simulated with only diffusion and production or diffusion and degradation, explaining why the AIC cannot predict the correct model in this case.

**Fig. 3:**
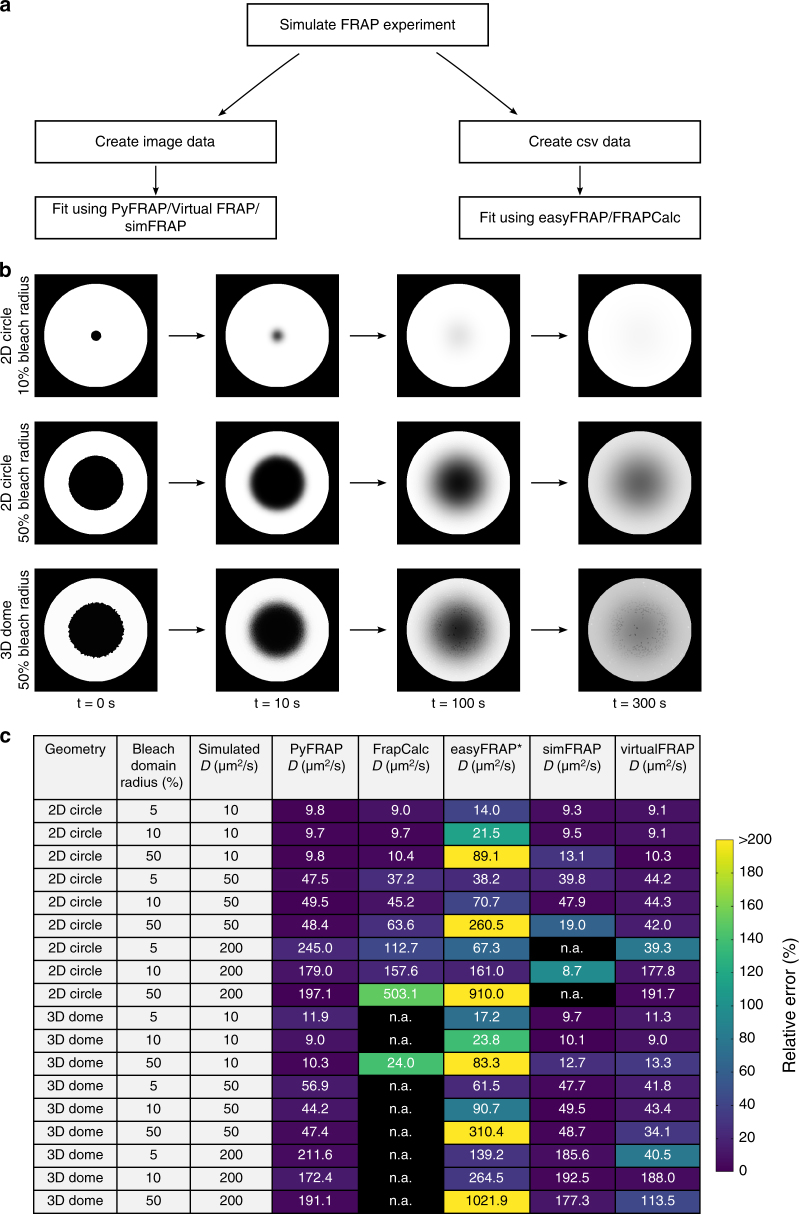
PyFRAP benchmarking simulation experiment. **a** Workflow of PyFRAP benchmarking. **b** Examples of simulated data sets for different bleach spot sizes and geometries. **c** Benchmarking results of PyFRAP against currently available software packages using simulated FRAP experiments. Simulation experiments varied in bleached region size, diffusion coefficient and experiment geometry. All diffusion coefficients and estimates are given in units of μm^2^/s. n.a. indicates that the software was not able to fit the simulated data. Colours indicate relative estimation error in %. Diffusion coefficients determined by easyFRAP (asterisk) were computed in combination with an equation providing a relationship between recovery rate, bleached domain size and diffusivity^45^

To assess PyFRAP’s performance in comparison with other available software packages based on analytical^17,46,47,59^ or numerical^20,21,60^ approaches (Supplementary Table 4), we used easyFRAP^47^, Virtual FRAP^20^, FrapCalc^46^, simFRAP^21^ and PyFRAP itself to analyse simulated FRAP experiments (Supplementary Note 2, Fig. 3). We simulated 18 experiments in which geometry, relative bleach window size, and diffusion coefficients differed. Simulations were conducted either in a simple circular two-dimensional domain or a complex three-dimensional zebrafish embryo-like geometry (Fig. 2e). FrapCalc and easyFRAP assume circular bleach windows^12,46,47^; to facilitate comparison, we therefore simulated FRAP experiments with circular bleach windows. Bleach window sizes comprised 5, 10 or 50% of the slice diameter, representing different proportions between fluorescent and bleached pools (Fig. 3b). Simulations were performed with three biologically relevant diffusion coefficients: 10, 50 and 200 μm^2^/s.

Simulation-based programs (PyFRAP, virtualFRAP and simFRAP) generally provided better results than analytical solutions (easyFRAP and FrapCalc): FrapCalc and easyFRAP were either unable to determine diffusion coefficients, or provided diffusivities that were off by at least 20% for most experiments (Fig. 3c). Fast recovery dynamics were challenging for all tested software. One reason for this is that fewer data points were recorded during the actual recovery process of highly diffusive molecules due to a fixed frame rate of 1 frame/s in the simulated test data sets, leading to larger errors; moreover, for fast recovery dynamics errors from interpolating simulations onto images are more severe. The analytical software packages provided better results for the two-dimensional compared to three-dimensional geometries, while simulation-based approaches showed no clear trend regarding geometry. In terms of bleach window radius, the analytical solutions performed worst if the window diameter was 50% of the slice diameter. This effect might be due to the assumption of an infinite pool of fluorescent molecules outside of the bleached region^12^—when the bleach window is very large, the pool of unbleached fluorescent molecules is small, which conflicts with the assumption of an infinite pool. In contrast, PyFRAP outperformed all current software packages and exhibited the smallest error between predicted and simulated diffusion coefficients (Fig. 3c).

### Applications of PyFRAP to measure diffusion hindrance

In vivo, it is thought that the overall movement of molecules is affected by binding interactions and by the presence of obstacles such as cells, resulting in a reduced effective diffusion coefficient of secreted proteins that move through tissues^22^. However, the effects of these interactions have not been rigorously tested experimentally. We therefore employed PyFRAP to examine the effects of obstacles and binding partners on the effective diffusivity of dextrans and proteins in experimentally controlled in vitro geometries and in living zebrafish embryos.

**Fig. 4:**
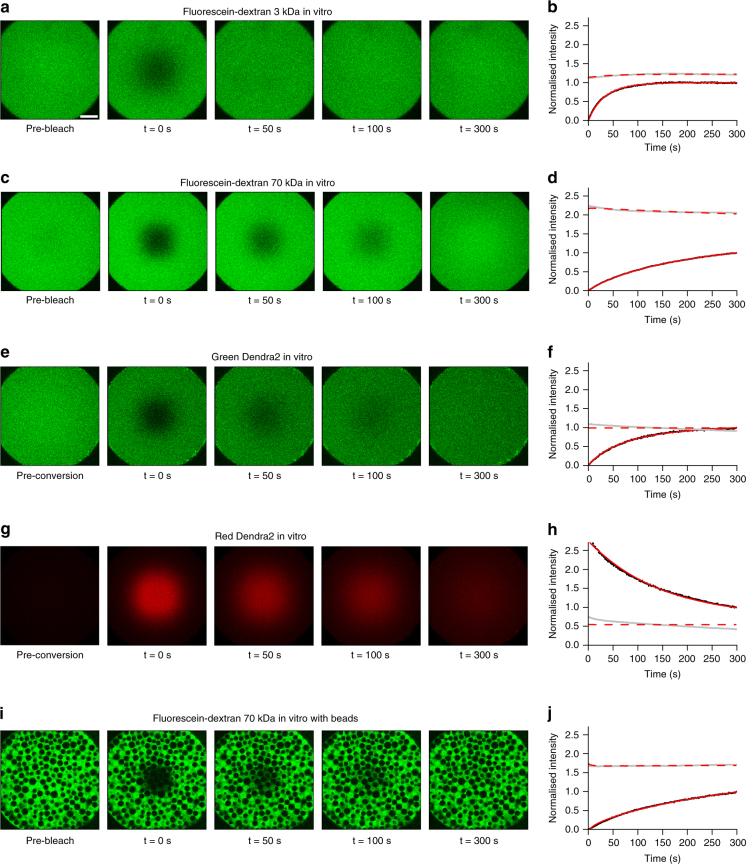
Examples of in vitro FRAP and iFRAP experiments and the resulting fits to measure free diffusion. **a**, **c**, **e**, **g**, **i** In vitro FRAP and iFRAP experiment images and **b**,** d**,** f**,** h**, **j** fits with PyFRAP. Black and grey dots represent data points of bleached and slice ROI, respectively. Red solid and dashed lines show the respective fits. **a**–**d** FRAP experiments with 3 and 70 kDa fluorescent dextrans (see Supplementary Fig. 5 for the full data set with fluorescent dextrans between 3 and 500 kDa). **e**–**h** iFRAP experiment with photoconverted Dendra2 protein showing data for the green (**e**,** f**) and the red (**g**,** h**) channel. **i**, **j** FRAP experiment with 70 kDa fluorescent dextran in the presence of polyacrylamide beads. Recovery curves were normalised between 0 (intensity in the bleached ROI at the first post-bleach time point) and 1 (intensity in the bleached ROI at the last post-bleach time point) to allow comparison across data sets. The length of the white scale bar in **a** represents 100 μm, and all images were acquired with the same magnification

**Fig. 5:**
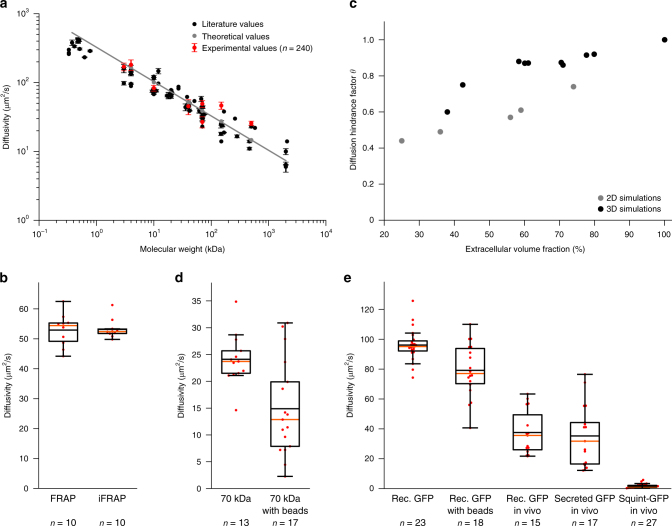
Effective diffusion coefficients determined by PyFRAP. **a** Results of in vitro experiments and PyFRAP analysis for freely diffusing fluorescent dextrans of different molecular weights. Black markers indicate literature values for fluorescent dextrans, red markers the mean effective diffusion estimates obtained by in vitro experiments and PyFRAP analysis, and grey markers the theoretical estimates derived from the Einstein-Stokes equation (see Supplementary Note 3). Red error bars show the standard deviation of PyFRAP’s effective diffusion estimates, and black error bars show the standard deviation of the literature values listed in Supplementary Table 7. The grey line represents a linear regression fit to the theoretical values. **b** Results of FRAP/iFRAP experiments for the photoconvertible protein Dendra2. **c** Results of simulations investigating the influence of tortuosity on effective diffusion for differently packed bead experiments. Grey and black markers indicate 2D and 3D simulation results, respectively. **d** Results of fluorescent dextran experiments demonstrating the impact of tortuosity on effective diffusivities. **e** Results of GFP experiments to analyse the impact of tortuosity, embryonic extracelluar environment, protein production, and extracellular binding on effective diffusion estimates. Box plots in **b**,** d**,** e** show median (orange line), mean (black horizontal line inside box), 25% quantiles (box), and all included data points (red markers). Whiskers extend to the smallest data point within the 1.5 interquartile range of the lower quartile, and to the largest data point within the 1.5 interquartile range of the upper quartile

First, we measured diffusion coefficients of a wide range of differently sized molecules (Supplementary Table 5) in a simple in vitro context in the absence of binding partners or obstacles. We performed FRAP experiments with different bleach geometries using fluorophore-coupled dextrans ranging from 3 to 500 kDa in molecular weight (Fig. 4a–d, Supplementary Figs. 5 and 6), and compared the results with theoretical predictions and literature values. Fluorescence recovery in these in vitro experiments should be purely defined by diffusion, and the theoretical diffusivities *D* of spherical molecules can be calculated from their radii *r* based on the relationship *D* ~ 1/*r* as postulated by the Einstein–Stokes equation (Supplementary Note 3). The diffusion coefficients determined by PyFRAP were in good agreement with literature values and theoretical predictions (Fig. 5a, Supplementary Tables 6 and 7).

A variant of FRAP that allows exclusion of reaction kinetics, such as production, and thus decrease the number of unknown experimental parameters is iFRAP (Fig. 1b). To perform in vitro iFRAP experiments, we used the green-to-red photoconvertible protein Dendra2^61^. Since photoconverting Dendra2 from green to red can also be interpreted as bleaching the original green fluorescence, measuring unconverted and converted protein distributions produces both FRAP and iFRAP experiments at the same time. To test whether PyFRAP correctly analyses iFRAP data, we used the experimental FRAP and iFRAP sets independently and assessed whether the obtained diffusion values are equal (Fig. 4e–h). Using FRAP we measured a Dendra2 diffusivity of 52.9 ± 5.2 (standard deviation) μm^2^/s, and using iFRAP we obtained a similar value of 53.3 ± 3.1 μm^2^/s (Fig. 5b, average difference between the two diffusivities per data set: 2.6 ± 1.5 μm^2^/s).

Next, we examined the effect of tortuosity on diffusion. In biological samples, the path length that molecules take increases as they move around obstacles such as cells. The effect of this tortuous movement can be described by the diffusion hindrance factor (also known as diffusion permeability^62^) *θ* = 1/*λ*^2^ = D^*^/*D*, where *λ* is the tortuosity, D^*^ is the effective diffusion coefficient (with obstacles), and *D* is the free diffusion coefficient (without obstacles). To assess the expected magnitude of tortuosity on altering effective diffusivity, we first performed numerical simulations of FRAP experiments with and without radial obstacles in two- and three-dimensional geometries. Radial obstacles were either placed regularly, randomly, or following a nearly-ideal packing scheme, resulting in an extracellular volume fraction (EVF, i.e., the space available for molecules to diffuse) ranging from 78% down to 25% (Supplementary Fig. 7). These simulations demonstrated that recovery rates are slowed down as the EVF decreases (Fig. 5c, Supplementary Table 8). If the geometry is two-dimensional, an EVF of 25% results in an expected reduction in effective diffusivity of approximately 66%. In three-dimensional simulation experiments, we obtained a reduction of effective diffusion coefficients by 40% when the EVF was decreased to 38% (Supplementary Note 3).

To determine whether the presence of obstacles decreases effective diffusivity as predicted by our simulations, we performed FRAP assays in vitro with a fluorescein-coupled 70 kDa dextran (Fig. 4i, j) or recombinant GFP (Supplementary Fig. 8) in the presence of polyacrylamide beads. Consistent with our predictions, recovery was slower in the presence of beads, and the effective diffusivity of fluorescein-coupled 70 kDa dextran dropped from 24.1 ± 0.4 (standard error) μm^2^/s to 14.9 ± 0.5 μm^2^/s, suggesting an EVF of 39% (*θ* = 0.61) (Fig. 5c, d, Supplementary Tables 8 and 9). Similarly, for recombinant GFP effective diffusivity dropped by 18% (Fig. 5e, Supplementary Table 10, Supplementary Fig. 8a–d).

To assess diffusion hindrance in vivo, we injected recombinant GFP protein into the extracellular space of living zebrafish embryos. We found that the effective diffusivity in vivo was 60% lower than for freely diffusing GFP, and 53% lower than in in vitro experiments with beads (Fig. 5e, Supplementary Table 10, Supplementary Fig. 8e, f). This suggests that tortuosity in zebrafish embryos is higher than in the in vitro bead assay. Importantly, we found similar diffusion coefficients of 36 μm^2^/s in vivo for extracellularly injected recombinant GFP and secreted GFP constantly produced from injected mRNA, showing that PyFRAP can properly account for both diffusion and production (Fig. 5e, Supplementary Table 10, Supplementary Fig. 8g, h).

Finally, we examined the effects of binding interactions on effective diffusivity. GFP presumably does not experience significant binding interactions with extracellular molecules in zebrafish embryos, although its movement is affected by obstructions like cells and cellular extensions. In contrast, secreted signalling molecules are expected to interact with extracellular molecules such as receptors and extracellular matrix components^22^. To assess the effect that interactions with extracellular molecules might have on secreted signalling molecules, we injected mRNA encoding the TGF*β*-superfamily member Squint fused to GFP into zebrafish embryos^29^. Squint-GFP is approximately 1.5 times larger than GFP and according to the Einstein-Stokes equation (Supplementary Note 3) would be predicted to have an approximately 1.14 times smaller diffusion coefficient than GFP (effective diffusivity *D*(GFP) = 36 μm^2^/s, expected effective diffusivity *D*(Squint-GFP) = 31 μm^2^/s). However, we measured an effective diffusion coefficient of approximately 2 μm^2^/s for Squint-GFP in living zebrafish embryos, ~90% lower than the predicted diffusion coefficient (Fig. 5e, Supplementary Table 10, Supplementary Figs. 8i, j and 9). These findings are consistent with previous measurements^29^ and with the idea that interactions with so far unidentified binding partners slow down the effective diffusion of embryonic signalling molecules like Squint-GFP^22,29^.

## Discussion

Although FRAP analyses have long been used to measure relative differences in mobilities between macromolecules, analysis tools to accurately and quantitatively determine effective diffusion coefficients from FRAP data are lacking. Current analysis tools impose several simplifications including one-dimensional or two-dimensional reductions of complex three-dimensional geometries, idealised bleaching conditions, and the absence of important reaction kinetics. When the experimental conditions closely resemble the simplified assumptions, e.g., small bleach domains and negligible reaction kinetics, these tools can rapidly provide reasonable diffusion estimates (Fig. 3c). However, experimental conditions are often more complex, and the use of simplified assumptions may yield drastically divergent diffusion coefficients (Fig. 1d–f). PyFRAP addresses these shortcomings by providing a simulation-based analysis that incorporates realistic geometries, bleaching conditions and reaction kinetics.

We found that PyFRAP’s data analysis pipeline is numerically reliable, recovered the correct diffusion coefficients and reaction kinetics, and additionally predicted the correct underlying reaction-diffusion models for simulated test data sets with known diffusion, production, and degradation parameters. PyFRAP consistently outperformed all other tested software packages, demonstrating its strength as a novel FRAP analysis method. Furthermore, PyFRAP was able to determine diffusion coefficients comparable to both theoretical and previously experimentally measured estimates for macromolecules with molecular weights ranging over two orders of magnitude. Since PyFRAP can analyse data independently of any assumptions about the initial conditions, it is suitable to analyse both FRAP and iFRAP experiments. iFRAP has recently been developed as an alternative to FRAP due the increasing availability of photoconvertible proteins and allows ignoring reaction kinetics such as production. We performed tandem FRAP/iFRAP experiments to analyse the diffusion of the photoconvertible protein Dendra2 and found equal diffusion coefficients in vitro with both methods.

FRAP experiments are typically performed in tissues in which macromolecules need to move around cellular obstacles, resulting in slower fluorescence recovery. To determine how this tortuosity might affect diffusion coefficients estimated from FRAP experiments, we first simulated FRAP experiments in two- and three-dimensional geometries introducing radial beads at different densities to vary the extracellular volume fraction (EVF). Our simulations showed a strong correlation between tortuosity and effective diffusivity and agree with previous theoretical work including Monte-Carlo simulations and homogenisation theory^62–65^. We then tested the predictions from these simulations with in vitro experiments using polyacrylamide beads to mimic cells. Compared to experiments without beads, the effective diffusion coefficient decreased by 39% (diffusion hindrance factor *θ* = 0.61) for 70 kDa fluorescein-dextran and 18% (*θ* = 0.82) for recombinant GFP. In living zebrafish embryos, effective diffusivity is much further reduced (Fig. 5e). It is unlikely that this is due to different viscosity of the extracellular medium in vivo, since free GFP diffusion is only marginally reduced in zebrafish embryos^22^. Instead, it is plausible that the complex geometries of real extracelluar environments—which include filopodia, extracellular matrix, and cavities that might act as dead end pores—could further increase tortuosity^62^. Finally, most in vivo FRAP experiments are affected by biochemical reactions such as production and degradation of proteins, which must be taken into account for accurate diffusion coefficient estimates (Fig. 1c, f). PyFRAP offers  various models for different reaction kinetics and can accurately estimate diffusion coefficients from data sets that include constant production and degradation.

PyFRAP measures effective diffusion, but due to its built-in PDE solver it could be extended in the future to consider spatially inhomogeneous kinetics and advective fluxes and to perhaps even determine the diffusivities of individual species in polydisperse mixtures of fluorescent molecules^66,67^. While PyFRAP can simulate three-dimensional FRAP experiments, FRAP data is currently almost exclusively obtained from two-dimensional confocal microscopy. In recent years, the development of light-sheet microscopy made fast three-dimensional imaging with low phototoxicity feasible^68^. In the future, PyFRAP’s image analysis tools could be extended to fit light-sheet microscopy data, which might provide deeper insights into the three-dimensional dynamics of molecule movement including convective flows or spatially inhomogeneous diffusion.

## Methods

### FRAP/iFRAP experiments in vitro

FRAP experiments to measure pure diffusion and tortuosity effects were conducted in a frustum-like plexiglass hole. Holes around 700 μm in diameter and about 100 μm in depth were drilled into a plexiglass block using a dental drill. Due to the small depth, the resulting shape was frustum-like with an upper base of 510 μm diameter.

Holes were filled with aqueous solutions of FITC-/fluorescein-labelled dextrans of different sizes, recombinant GFP, or Dendra2 protein (Supplementary Table 5) using a micro-pipette. Dendra2 protein was centrifuged at 16,000 × *g* for 30 min at 4 °C to remove protein aggregates. Excess liquid was removed from the hole by pipetting under observation with a stereo microscope.

To model the effect of tortuosity in the in vitro FRAP experiments, polyacrylamide beads were added to the sample solution. The microbeads (Bio-Gel P-2 Gel, <45 μm wet bead size) were first soaked in distilled water overnight for hydration. The beads were then centrifuged at 300 × *g*, the supernatant removed, and the required quantity of beads transferred to another tube for resuspension in fluorescein-dextran or GFP+BSA solution. This was repeated and followed by removal of the supernatant, leaving a concentrated slurry of beads and fluorescent solution for the experiments. The beads were transferred into the plexiglass template and settled within 1–2 min.

To prevent evaporation, mineral oil (Sigma) was placed around the solution before sealing the hole with a cover slip (No 1.5). Supplementary Fig. 1a outlines the sample preparation process for in vitro experiments. The sample was upended carefully and mounted on an inverted confocal microscope. Images were taken using an LSM 780 NLO microscope (ZEISS) with an LD LCI Plan-Apochromat 25×/0.8 Imm Korr DIC objective (ZEISS) and immersion oil (Immersol TM W, *n* = 1.334 at 23 °C, ZEISS). First, a plane approximately in the middle of the hole was chosen and the *z*-position set to zero. Then, the position of the highest and lowest point was determined. Cuboid volumes (141.42 μm × 141.42 μm × 100 μm) were bleached by imaging a *z*-stack at highest laser power (488 nm) or photoconverted at moderate laser power. Time series of 300 images (512 pixels × 512 pixels) were taken with a speed of 1 frame/s (pixel dwell time: 3.15 μs) over a duration of 5 min. The zoom was set to 0.7, and the resulting images had a size of 566.79 μm × 566.79 μm.

After the FRAP experiment, the template was cleaned using distilled water, soap, and an interdental toothbrush.

### FRAP experiments in vivo

Zebrafish embryos (*Danio rerio*) were collected 10 min after mating and proteolytically dechorionated^22,29,42^. For the experiments with recombinant GFP, 100 pg of recombinant GFP were injected into the extracelluar space when zebrafish embryos reached high stage^22,29,69^ (Supplementary Table 10). For experiments with secreted GFP^29^, 100 pg of the mRNA encoding the fluorescent protein were injected at the one-cell stage. For experiments with Squint-GFP^29^, either 30 or 200 pg of mRNA were injected at the one-cell stage. At dome stage, embryos were mounted in drops of 1% low-melting-point agarose animal pole down onto a glass-bottom dish (MatTek Corp. P35G-1.5-20-C), and as soon as the drops solidified covered with Danieau’s medium^29,42^ to prevent the embryos from drying out. Supplementary Fig. 1b outlines the in vivo sample preparation process.

Confocal images were taken roughly at a depth of 40 μm from the animal pole into the embryo. For data sets injected with 200 pg of Squint-GFP-encoding mRNA, images were acquired with the same settings as described for the in vitro experiments either with 1 frame/s for 300 s, or 1 frame/10 s for 3000 s. Images of embryos injected with 30 pg of Squint-GFP-encoding mRNA were taken with a spatial resolution of 340.08 μm × 340.08 μm and 1 frame/10 s for 3000 s. Data sets for recombinant GFP in vivo were acquired with the same microscope settings as the experiments conducted in vitro.

### ROI selection

PyFRAP’s image analysis depends on defining specific ROIs for the experimental data and simulations. Users can define multiple different geometrical shapes of ROIs in three-dimensional space such as cylinders, prisms, and any kind of addition or subtraction between ROIs. The specified ROIs are then used for image analysis, estimating concentrations outside the field of view, evaluating the simulation, and fitting to the analysed data. PyFRAP is equipped with an ROI manager and wizards for several standard sets of ROIs.

### Image analysis

Let Ω_*i*_ (with *i* ∈ {1, 2, …, *n*_Ω_} and *n*_Ω_ the number of ROIs) be the list of ROIs specified for PyFRAP’s analysis. The mean intensity over the ROI Ω_*i*_ at time *t*_*j*_ (with *j* ∈ {1, 2, …, *n*_*t*_} and *n*_*t*_ the number of images) is then calculated by

1 $$\bar I_{{\mathrm{\Omega }}_i}\left( {t_j} \right) = \frac{1}{{A_i}}\mathop {\sum}\limits_{\left( {x_k,y_l} \right) \in {\mathrm{\Omega }}_i} I\left( {x_k,y_l,t_j} \right)$$

where *A*_*i*_ is the area of Ω_*i*_, and *I*(*x*_*k*_, *y*_*l*_, *t*_*j*_) is the intensity at pixel (*x*_*k*_, *y*_*l*_) (with *k* ∈ {1, 2, …, *n*_*x*_} and *n*_*x*_ the number of rows in the images, and with *l* ∈ {1, 2, …, *n*_*y*_} and *n*_*y*_ the number of columns in the images).

FRAP image data were analysed within the ROIs Ω_bleached_ and Ω_slice_. Ω_slice_ was defined as a circular domain with centre *C*_slice_ and radius *r*_slice_. Since the imaging depth varied between experiments, both *C*_slice_ and *r*_slice_ were cropped for each data set. The bleached ROI Ω_bleached_ was defined as a square with sidelength *s*_bleached_ and left-lower corner at *O*_bleached_ = *C*_slice_ − $\frac{1}{2}$(*s*_bleached_, *s*_bleached_). The definition of both ROIs is shown in Supplementary Fig. 2a.

### Accounting for uneven illumination

Uneven imaging due to inhomogeneous sample illumination is a common problem in microscopy^37–39^. We implemented two solutions in PyFRAP to address this problem: (1) Normalisation by an image acquired before bleaching, and (2) applying a flattening mask derived from imaging a homogeneous fluorescent sample. The pixel-wise mean image over *n*_t_ images can be defined as

2 $$M\left( {x_k,y_l,t_j} \right) = \frac{1}{{n_{\mathrm{t}}}}\mathop {\sum}\limits_{j = 1}^{n_{\mathrm{t}}} {\kern 1pt} I\left( {x_k,y_l,t_j} \right)$$

To avoid noise-induced singularities when normalising, PyFRAP computes a mean normalisation mask *M*_pre_ over multiple pre-bleach images, and then divides each image of the recovery time series pixel-wise by the computed mask

3 $$\tilde I\left( {x_k,y_l,t_j} \right) = \frac{{I\left( {x_k,y_l,t_j} \right) + O_{{\mathrm{norm}}}}}{{M_{{\mathrm{pre}}}\left( {x_k,y_l} \right) + O_{{\mathrm{norm}}}}}$$

where *O*_norm_ is the optimal data offset computed via

4 $$O_{{\mathrm{norm}}} = {\mathrm{max}}\left\{ {\mathop {{{\mathrm{min}}}}\limits_{k,j} \left( {I\left( {x_k,y_l,t_j} \right)} \right),\mathop {{{\mathrm{min}}}}\limits_{k,j} \left( {M_{{\mathrm{pre}}}\left( {x_k,y_l,t_j} \right)} \right)} \right\} + 1$$

Similarly, the flattening mask *F* is computed using the mean over multiple images of a fluorophore spread homogeneously across a cover slip, *M*_flat_:

5 $$F\left( {x_k,y_l} \right) = \frac{{{\mathrm{max}}_k\left( {M_{{\mathrm{flat}}}\left( {x_k,y_l} \right)} \right) + O_{{\mathrm{flat}}}}}{{M_{{\mathrm{flat}}}\left( {x_k,y_l} \right) + O_{{\mathrm{flat}}}}}$$

Similar to the normalisation in Eq. (4), the optimal data offset *O*_flat_ is obtained by taking the maximum over all minimum intensities of images in both recovery and flattening data sets. The recovery data set is obtained by pixel-wise multiplication of the recovery image with the flattening mask obtained in Eq. (5):

6 $$\tilde I\left( {x_k,y_l,t_j} \right) = F\left( {x_k,y_l} \right) \cdot I\left( {x_k,y_l,t_j} \right)$$

An outline of both correction methods is shown in Supplementary Fig. 2c.

In the present study, two pre-bleach images were acquired per sample for the normalisation mask, and two images of fluorescein conjugated to a 40 kDa dextran or recombinant GFP homogeneously spread on a cover slip were acquired for the flattening approach. The effects of flattening and normalisation on data analysis are described in Supplementary Note 1.

### Accounting for background fluorescence

Background subtraction is a standard procedure to extract the true signal of microscope images^38,39^. Similar to the flattening and normalisation masks, PyFRAP takes the average over multiple pixels to obtain a background mask and then subtracts it pixel-wise^38,39^:

7 $$\tilde I\left( {x_k,y_l,t_j} \right) = I\left( {x_k,y_l,t_j} \right) - M_{{\mathrm{bkgd}}}\left( {x_k,y_l} \right)$$

The mean of two images without a sample was determined to compute a background mask. The effect of background subtraction is discussed in Supplementary Note 1.

### Application of filters for noise reduction

Microscope data sets are often noisy, causing problems for normalisation and simulation. PyFRAP smooths noisy pixels by either applying a Gaussian blur with standard deviation *σ*_gauss_, or a median filter with filter window radius *r*_median_. We found that *σ*_gauss_ = 2 and *r*_median_ = 5 provided good results for the data in the present study (see Supplementary Note 1).

### Accounting for fluorescence outside of the imaging view

In some cases it is not possible to capture the whole sample in one field of view under the microscope, and the concentration in the non-imaged regions needs to be estimated. PyFRAP solves this by letting users define an ROI Ω_rim_ to select an approximation of the average unbleached intensity from the first image of the recovery image series:

8 $$c_{{\mathrm{rim}}} = \frac{1}{{A_{{\mathrm{rim}}}}}\mathop {\sum}\limits_{\left( {x_k,y_l} \right) \in {\mathrm{\Omega }}_{{\mathrm{rim}}}} I\left( {x_k,y_l,t_0} \right)$$

Ω_rim_ is defined by Ω_rim_ = Ω_slice_ − Ω_centre_, where

9 $${\mathrm{\Omega }}_{{\mathrm{center}}} = \left\{ {\left( {x_k,y_l} \right)|\sqrt {\left( {x_k - x_c} \right)^2 + \left( {y_l - yc} \right)^2} < \rho _{{\mathrm{rim}}}r_{{\mathrm{slice}}}} \right\}$$

with (*x*_*c*_, *y*_*c*_) the centre pixel coordinates of the image. Ω_rim_ thus defines a small annulus comprising all pixels (*x*_*k*_, *y*_*l*_) inside Ω_slice_ that have a distance of at least *ρ*_rim_*r*_slice_ from the centre of the image (Supplementary Fig. 2b). *ρ*_rim_ = 0.66 and *ρ*_rim_ = 0.4585 were found to provide good values for the in vitro and in vivo experiments, respectively.

### Simulations

PyFRAP simulates FRAP experiments numerically. Ignoring reaction kinetics, a FRAP experiment can be described by the diffusion equation

10 $$\frac{{\partial c({\bf{x}},t)}}{{\partial t}} = D\nabla ^2c({\bf{x}},t),{\bf{x}} \in {\mathrm{\Omega }}$$

where *c*(**x**, *t*) is the concentration of the measured molecule at position **x** = $\left\langle {x,y,z} \right\rangle$ and time *t* inside the domain Ω, and *D* is its scalar diffusion coefficient. The diffusion coefficient is assumed to be constant and homogeneous.

Since the sample is assumed to be a closed system, no-flux Neumann boundary conditions were defined as

11 $$\frac{{\partial c({\bf{x}},t)}}{{\partial {\bf{n}}}} = {\mathrm{0}},{\bf{x}} \in \partial {\mathrm{\Omega }}$$

where **n** is the normal vector of the boundary ∂Ω at position **x**.

### Initial conditions for simulations

The initial conditions are given by the bilinear interpolation *P* between pixels of the initial post-bleaching image:

12 $$P(x,y) = \frac{{\left( {x_2 - x,x - x_1} \right)}}{{\left( {x_1 - x_2} \right)\left( {y_2 - y_1} \right)}} \cdot \left( {\begin{array}{*{20}{c}} {I\left( {x_1,y_1} \right)} & {I\left( {x_1,y_2} \right)} \\ {I\left( {x_2,y_1} \right)} & {I\left( {x_2,y_2} \right)} \end{array}} \right) \cdot \left( {\begin{array}{*{20}{c}} {y_2 - y} \\ {y - y_1} \end{array}} \right)$$

*I*(*x*_*k*′_, *y*_*l*′_) with *k*′, *l*′ ∈ {1, 2} represents the intensities in the initial image of the four pixels surrounding (*x*, *y*). If (*x*, *y*) is outside of the visible ROI in the initial image (Ω_1_), the rim concentration *c*_rim_ given in Eq. (8) is combined piece-wise with Eq. (12) to give the initial condition

13 $$c({\bf{x}},0) = \left\{ {\begin{array}{*{20}{l}} {P(x,y)} \hfill & {{\mathrm{if}}\left( {x,y} \right) \in {\mathrm{\Omega }}_1\forall z} \hfill \\ {c_{{\mathrm{rim}}}} \hfill & {{\mathrm{otherwise}}} \hfill \end{array}} \right.$$

### Simulation geometry

PyFRAP comes with its own geometry definition tool. Geometry definitions can then be converted into the Gmsh format^43^ for meshing. PyFRAP can read Gmsh’s geometry definition files, use Gmsh’s mesh files, or import STereoLithography (.stl) files, allowing users to define arbitrary two- and three-dimensional geometries. This gives users the ability to describe a realistic FRAP experiment geometry with the necessary precision.

The simulation geometry Ω for the in vitro experiments was a conical frustum with upper radius *r*_upper_ = 317.65 pixels, lower radius *r*_lower_ = 224.25 pixels, and height *h* ≈ 90.33 pixels (Supplementary Fig. 4b). For the in vivo experiments, the simulation geometry resembled a zebrafish embryo at dome stage, i.e., the intersection of two hemispheres intersecting each other at the equator of the outer hemisphere. Since the geometry depends on the radius of the embryo in the initial image, *r*_imaging_ was calculated separately for each experiment^29,70^. Assuming that the radius of the inner hemisphere *r*_inner_ is 10% larger than the one of the outer hemisphere, *r*_outer_, the geometry can be computed by

14 $$\begin{array}{l}r_{{\mathrm{outer}}} = \frac{{r_{{\mathrm{imaging}}}^2 + h_{{\mathrm{imaging}}}^2}}{{ - 2h_{{\mathrm{imaging}}}}}\\ r_{{\mathrm{inner}}} = 1.1 \cdot r_{{\mathrm{outer}}}\\ d_{{\mathrm{center}}} = \sqrt {r_{{\mathrm{inner}}}^2 - r_{{\mathrm{outer}}}^2} \end{array}$$

where *d*_centre_ is the distance between the two centres of the hemispheres. Supplementary Fig. 4a shows a schematic of the zebrafish dome stage geometry.

### Meshing for simulations

PyFRAP discretises simulation geometries using Gmsh^43^ in combination with TetGen^44^ into tetrahedral meshes. PyFRAP utilises almost all functionalities of Gmsh—such as boundary layer meshes, attractor meshes, mesh merging and mesh refinement—allowing users to apply fine meshes where they are needed.

The overall default element size in the present study was *v* = 25 pixels^3^. To overcome numerical instabilities, such as Gibbs phenomena at the boundary of Ω_bleached_, the mesh around the bleached area boundary was refined using a boundary layer mesh of thickness *w*_BL_ = 30 pixels and element size *v*_BL_ = 15 pixels^3^. Since only the simulation inside Ω_slice_ and Ω_bleached_ is used to fit the FRAP experiments, the mesh inside Ω_slice_ was also refined to an element size of *v*_slice_ = 15 pixels^3^. Supplementary Fig. 4c, e shows an example of a tetrahedral mesh with both slice refinement and boundary layer meshes for the zebrafish dome geometry described in the previous section.

### PDE solver

All partial differential equations (PDEs) were simulated using the FiPy toolbox^32^. The LU factorisation algorithm or the Preconditioned-Conjugated-Gradient algorithm implemented in PySparse were used to solve the linear system at each time step.

### Simulation parameters

All simulations were performed with a reference diffusion coefficient of *D* = 50 pixels^2^/s. To ensure that the simulations run long enough to capture the full recovery of the FRAP experiment, the end time point of the simulation was set to *t*_sim,end_ = 1680 s for experiments conducted with an acquisition interval of Δ*t* = 1 s. Since the recovery is steepest at the beginning of the simulations, a logarithmic time-stepping scheme was used, making early time steps shorter to achieve greater accuracy. A summary of all simulation parameters used to analyse the FRAP data in the present study is given in Supplementary Table 11.

### Fitting

To avoid the need to re-simulate the FRAP experiment for each choice of diffusion coefficient *D*, PyFRAP uses the self-similarity property of the solution to Eq. (10). For example, a simulated FRAP experiment with the diffusion coefficient *D* = 50 pixels^2^/s results in the same recovery behaviour as an experiment with the diffusion coefficient *D* = 200 pixels^2^/s, just four times slower. This can be described as

15 $$c({\bf{x}},t,D) = c\left( {{\bf{x}},\frac{{D_{{\mathrm{ref}}}}}{D}t,D_{{\mathrm{ref}}}} \right)$$

where *D*_ref_ is the reference diffusion coefficient, i.e., the diffusion coefficient used for the simulation of Eq. (10). Supplementary Fig. 4d shows simulated recovery curves for various diffusion coefficients illustrating this self-similarity property.

PyFRAP allows users to fit four different models to FRAP data: (1) Pure diffusion, (2) diffusion and production, (3) diffusion and degradation, (4) diffusion with degradation and production, and each of these models with an additional set of equalisation parameters (see below). In case of pure diffusion, the solution for the diffusion coefficient *D* over a given ROI Ω_*i*_ is simply given by the volume integral of the solution in Eq. (15):

16 $$\tilde c\left( {{\mathrm{\Omega }}_i,t,D} \right) \equiv \mathop {\int}\limits_{{\bf{x}} \in {\mathrm{\Omega }}_i} {\kern 1pt} c({\bf{x}},t,D){\mathrm{d}}V$$

A summary of all parameters used to fit the FRAP data in the present study is given in Supplementary Table 12.

### Extending the diffusion model with reaction kinetics

Spatially uniform production was added to the scaled FRAP model defined in Eq. (15) or in Eq. (20) by

17 $$\bar c({\mathrm{\Omega }}_i,t,D) = c(\Omega _i,t,D) + k_2t$$

where *k*_2_ is the production rate. To add spatially uniform degradation, the resulting solution is given by

18 $$\bar c\left( {{\mathrm{\Omega }}_i,t,D} \right) = c\left( {\Omega _i,t,D} \right)e^{ - k_1t}$$

The parameter *k*_1_ represents the degradation rate constant. Adding both degradation and production to the system results in the following superposition of solutions:

19 $$\bar c({\mathrm{\Omega }}_i,t,D) = c({\mathrm{\Omega }}_i,t,D)e^{ - k_1t} + \left( {1 + e^{ - k_1t}} \right)\frac{{k_2}}{{k_1}}$$

### Accounting for varying fluorophore fractions by equalisation

FRAP experiments can vary in intensity during the experiment due to, for example, an increase or decrease in extracellular volume fraction, due to molecules moving in and out of the imaging plane, or due to an immobile fraction of fluorescent molecules. These effects are accounted for by equalisation, which normalises both simulation and data recovery curves to an equivalent scale between 0 and 1. During the fitting process, the simulated recovery curves are slightly lifted or lowered to better resemble overall fluorescence levels. This can be written as

20 $$\tilde c({\mathrm{\Omega }}_i,t,D) = \frac{1}{{\bar c_{{\mathrm{max}}}E_i}}\left( {\mathop {\int}\limits_{{\bf{x}} \in {\mathrm{\Omega }}_i} {\kern 1pt} \bar c({\bf{x}},t,D)\left. {{\mathrm{d}}V - \bar c_{{\mathrm{min}}}} \right)} \right)$$

where *E*_*i*_ is the equalisation factor for ROI Ω_*i*_. The background $\bar c_{{\mathrm{min}}}$ was chosen to be the smallest concentration of the bleached ROI inside the imaging region (Ω_bleached_), over the whole time series

21 $$\bar c_{{\mathrm{min}}} = \mathop {{{\mathrm{min}}}}\limits_t \mathop {\int}\limits_{{\bf{x}} \in {\mathrm{\Omega }}_{{\mathrm{bleached}}}} {\kern 1pt} \bar c({\bf{x}},t){\mathrm{d}}V$$

and the normalisation value $\bar c_{{\mathrm{max}}}$ to be the maximum concentration inside the whole imaging ROI (Ω_slice_), over the whole time series

22 $$\bar c_{{\mathrm{max}}} = \mathop {{{\mathrm{max}}}}\limits_t \mathop {\int}\limits_{{\bf{x}} \in {\mathrm{\Omega }}_{{\mathrm{slice}}}} {\kern 1pt} \bar c({\bf{x}},t){\mathrm{d}}V$$

### Minimisation and parameter estimation

Choosing one of the models defined in Eqs. (15), (16), (17), (18) and (19), the sum of squared differences, SSD, was calculated by

23 $$\mathrm{SSD} = \mathop {\sum}\limits_i {\kern 1pt} \mathop {\sum}\limits_{t_j} \left( {\tilde c\left( {{\mathrm{\Omega }}_i,t_j,D} \right) - I_{{\mathrm{\Omega }}_i}\left( {t_j} \right)} \right)^2$$

where *t*_*j*_ ∈ 0, .., *T* are all time points of the FRAP data set, and Ω_*i*_ ∈ Ω_bleached_, Ω_slice_ are the two ROIs of interest yielding a mean optimal fit between all fitted ROIs. The minimisation of Eq. (23) was carried out using a constrained Nelder–Mead algorithm^49^. Since especially for a larger number of degrees of freedom the minimisation algorithm tended to stop in local minima, initial guesses for the diffusion coefficient *D* were tested over two orders of magnitude, and the fit yielding the minimum SSD was considered optimal.

### Analysis speed

Details of the method to determine PyFRAP’s performance in terms of analysis speed are described in Supplementary Note 4 and Supplementary Tables 13 and 14.

### Statistics

PyFRAP offers four statistical tools (Supplementary Table 2) allowing users to test whether the estimated diffusion coefficient for one experimental group is significantly different from another one. The statistical tools include the two most prominent parametric significance tests, the Student’s *t*-test^56^ and a modification of this test, Welch’s *t*-test^71^, which both assume normally distributed test groups. PyFRAP also provides the Shapiro–Wilk test, allowing PyFRAP users to quickly assess whether the estimated diffusion coefficients follow a normal distribution. The Shapiro–Wilk test was recently found to have the best sensitivity compared to other common normality tests^72^. If normality cannot be guaranteed, PyFRAP offers two non-parametric ranked hypothesis tests: The Wilcoxon signed-rank test^73^ and the Mann–Whitney U test^57^.

Often, the underlying reaction kinetics of FRAP experiments or the relevance of their contribution might be unknown^54^. However, models with more parameters generally provide better fits than simpler models. The AIC^55^ allows users to evaluate which model fits the data the best while keeping model complexity low. For this, let

24 $${\mathrm{\Theta }}: = \left( {k_1,k_2,D,E_1,E_2, \ldots } \right)$$

be the vector of unknown diffusion coefficient *D*, reaction rates *k*_1_ and *k*_2_, and *E*_1_, *E*_2_, … a list of equalisation factors. Moreover, let *m* = *m*(Θ) be the model prediction using Θ. Assuming that the data is distributed normally around the model

25 $$d_i - m_i\sim {\cal N}(\mu ,\sigma)$$

the log-likelihood function at data point *i*, *L*_*i*_ becomes

26 $$L_i\left( {{\mathrm{\Theta }}|d_i - m_i} \right) = \left( {d_i - m_i} \right)^2$$

and is thus identical with the sum of squared differences used for optimisation in Eq. (23):

27 $$L({\mathrm{\Theta }}) = \mathop {\sum}\limits_i {\kern 1pt} L_i({\mathrm{\Theta }}) = \mathrm{SSD}$$

The AIC is then given by

28 $$\mathrm{AIC} = 2k - 2L\left( {{\hat{\mathrm\Theta }}} \right)$$

where *k* is the number of parameters of model *m* and

29 $$\hat \Theta = {\mathrm{argmin}}(L(\Theta |d_i - m_i,i = 1...n))$$

is the parameter configuration Θ minimising the log-likelihood function (Eq. (27)), i.e., the parameter configuration returned from fitting the model to data. The best model according to the AIC is then *m*(argmin(AIC_*i*_ − AIC_min_)). If the number of sample points is small, the corrected AIC (AICc) provides a more accurate model selection technique:

30 $$\mathrm{AICc} = \mathrm{AIC} + \frac{{2k(k + 1)}}{{n - k - 1}}$$

where *n* is the number of data points. A rule of thumb for when the AIC (Eq. (28)) or its corrected version (Eq. (30)) should be used is

31 $$\frac{n}{k} > 40$$

PyFRAP automatically selects which statistical model is more appropriate if not specified differently.

PyFRAP also provides *R*^2^-values for each fit: An *R*^2^-value for each fitted ROI and the product and mean of these values. In general, PyFRAP computes an *R*^2^-value of an ROI by

32 $$R^2 = 1 - \frac{{\mathop {\sum}\limits_i {\kern 1pt} m_i - d_i}}{{\mathop {\sum}\limits_i {\kern 1pt} d_i - \bar d}}$$

where *m*_*i*_ and *d*_*i*_ are model and data at time *i*, and $\bar d$ is the mean over all data points.

### Data exclusion

We performed a rigorous screen of all data sets, and we excluded data sets that showed strong radial inhomogeneities in the first post-bleach image due to inhomogeneous distribution of fluorescent molecules. Moreover, we excluded in vitro data sets that showed unstable distributions in the overall fluorescence intensity levels, indicating incomplete bleaching through the depth of the sample.

### Code availability

PyFRAP is freely available from https://mueller-lab.github.io/PyFRAP.

### Data availability

All data is available from the corresponding author upon request.

## Electronic supplementary material

Supplementary Information
