# Supplementary material for: Quantitative diffusion measurements using the open-source software PyFRAP
Source: Nat Commun. 2018 Apr 20;9:1582. doi: 10.1038/s41467-018-03975-6 (PMC5910415; doi:10.1038/s41467-018-03975-6)
Supplement: Supplementary file 1 — Supplementary Information [file 41467_2018_3975_MOESM1_ESM.pdf]

Supplementary Information for

Quantitative diffusion measurements using the open-source software PyFRAP

Alexander Bläßle[1], Gary Soh[1], Theresa Braun[1,2], David Mörsdorf[1], Hannes Preiß[1], Ben M. Jordan[3], and Patrick Müller[1,*]

[1]Friedrich Miescher Laboratory of the Max Planck Society, Tübingen, Germany
[2]Present address: University of Konstanz, Konstanz, Germany
[3]Department of Organismic and Evolutionary Biology, Harvard University, Cambridge, USA

[*]Correspondence to: patrick.mueller@tuebingen.mpg.de (P.M.)

This PDF includes:
Supplementary Notes 1-4
Supplementary Tables 1-14
Supplementary Figures 1-9
Supplementary References 1-43

Supplementary Note 1: Analysis method selection

To assess how image correction by flattening, normalisation, background subtraction, Gaussian blur, and median filter application affects effective diffusion estimates from FRAP experiments, we tested the 24 analysis combinations listed in Supplementary Table 1. We assessed whether 1) the resulting diffusion estimate D is affected, 2) the standard deviation σ of the estimated diffusion coefficients is affected (i.e. whether correcting and smoothing the images makes the diffusion estimates more exact), and 3) the goodness of the fits (i.e. R^2-values) is affected. We quantified the effect of an analysis option by

$$H(v, \alpha) = \frac{v(\alpha)}{v(\alpha_0)} \tag{1}$$

where $\alpha = \{n, f, b, g, m\}$ represents an analysis option defined by five binary entries indicating whether normalisation n, flattening f, background subtraction b, Gaussian blur g, or a median filter m was used. If we did not correct images, we denote this by α_0. The variable v describes the quantified result, such as the mean diffusion coefficient. Supplementary Fig. 3 shows a subset of this analysis for three different variables v: The mean diffusion coefficient D, the variance of diffusion coefficients σ, and the fit quality R^2. FRAP experiments performed in the present study were grouped by condition (*in vitro* experiments with free diffusion, *in vitro* experiments with beads, and *in vivo* experiments) to isolate condition-specific effects. We did not correct for potential illumination inhomogeneities in *in vivo* experiments, since these only covered a small centered area of the total image, and illumination is homogeneous in this region. Moreover, normalisation cannot be used for the analysis of *in vitro* experiments containing beads, since normalisation would introduce artificially high intensity areas at the locations of the beads.

If only normalisation and flattening were applied, we observed an increase of the apparent diffusion coefficients and an improvement in fit quality for free diffusion (Supplementary Fig. 3a,c). Both techniques only mildly affected the variance of diffusion coefficients (Supplementary Fig. 3b). Background subtraction had no effect on any measure (Supplementary Fig. 3a-c). Moreover, noise reduction or smoothing via median filter or Gaussian blur application tended to decrease the variance in all conditions (Supplementary Fig. 3b) and improve the fits for free diffusion (Supplementary Fig. 3b).

We also tested whether a combination of an illumination correction technique (n or f) with the remaining three manipulation techniques (b, g and m) can further improve the analysis. Supplementary Fig. 3d shows that this can lead to an increase in mean apparent diffusion estimates similar to those observed in Supplementary Fig. 3a. Moreover, applying a median filter or Gaussian blur in combination with flattening improves fit quality and decreases diffusion estimate variance (Supplementary Fig. 3e,f).

To keep the extent of image manipulation as minimal as possible while obtaining comparable low-variance estimates from high-quality fits, we only applied flattening to correct the images from *in vitro* experiments. Since both Gaussian blur and median filter treatments appeared to stabilise diffusion coefficient estimates (i.e. reducing their variance) to a similar extent, we restricted image smoothing to the application of a median filter for all other analyses.

Supplementary Note 2: Comparison of PyFRAP to other FRAP analysis software

We selected four current FRAP analysis software packages for comparison with PyFRAP: The two analytical programs easyFRAP[1] and FrapCalc[2], and the two numerical packages virtualFRAP[3] and simFRAP[4] (Supplementary Table 4).

To assess the performance of PyFRAP in comparison with other FRAP analysis software solutions, we created simulated FRAP data sets using PyFRAP's simulation toolbox. We found that PyFRAP and our in-house software based on MATLAB and COMSOL Multiphysics[5–7] produced identical simulated data, and we subsequently chose to use PyFRAP to simulate the experiments due to the ease of PyFRAP's scripting abilities. We simulated two-dimensional or three-dimensional FRAP experiments with circular bleaching spots of various sizes for a 300 s time-course. Two-dimensional simulated experiments were conducted in a circle with radius 215 µm, and three-dimensional experiments resembled a zebrafish at dome stage with $r_{\mathrm{imaging}} = 215$ µm and $h_{\mathrm{imaging}} = 80$ µm (see Methods section for details). Molecules were allowed to move with diffusion coefficients of 10 µm^2/s, 50 µm^2/s, or 200 µm^2/s, covering a range of typical diffusivities in biological samples. Bleached spots were placed in the center of the simulation geometry and comprised 5%, 10%, or 50% of the slice radius. We chose the boundary layer mesh described in the Methods section to envelope the bleached spot, guaranteeing numerical accuracy of the simulation experiments. PDEs were simulated over 4000 logarithmically-spaced time steps. The simulations were saved in a csv sheet specifically formatted for the use of easyFRAP or FrapCalc, or in 301 images by interpolation of the numerical solution onto a 512 µm $\times$ 512 µm grid. We then either imported and analysed the csv sheet using FrapCalc (`https://github.com/miura/FrapCalc` for IgorPro7) or easyFRAP, or read in and analysed the simulated images using simFRAP or virtualFRAP. The benchmarking analysis was performed using Microsoft Windows 8.1.

In contrast to other programs that determine absolute diffusion coefficients, easyFRAP only provides recovery half times ($1/\tau_{\frac{1}{2}}$). Thus, to compute diffusion coefficients from easyFRAP, we used the well-established[8] equation

$$D = \frac{-\omega^2 \ln\left(\frac{1}{2}\right)}{\tau_{\frac{1}{2}}}$$

with various dimensions of the bleached spot ω.

We used PyFRAP's standard pipeline to analyse the saved simulated FRAP images files in an unbiased manner, only constraining imaging depth and radius.

As mentioned in the main text, PyFRAP outperformed all tested software packages and exhibited the smallest error between predicted and simulated diffusion coefficients (Fig. 3c).

Supplementary Note 3: Data analysis and control experiments

Computation of theoretical diffusion coefficients

We compared our *in vitro* FRAP results for differently sized fluorescein-labeled dextrans to predictions derived from the Einstein-Stokes equation

$$D = \frac{k_B T}{6\pi\eta r} \tag{2}$$

where $k_B = 1.380\,648\,52 \times 10^{-23}$ $m^2\,kg\,s^{-2}\,K^{-1}$ is the Boltzmann constant. The FRAP experiments were conducted in an aqueous solution with viscosity $\eta = 0.9321 \times 10^{-3}$ $kg\,s^{-1}\,m^{-1}$ at $T = 296$ K. Stokes radii r of the fluorescent molecules were obtained from the manufacturers' websites and are listed along with the calculated theoretical diffusion coefficients in Supplementary Table 5.

FRAP experiments with different bleach window sizes

To test whether different bleach window size might lead to different diffusion coefficient estimates, we performed FRAP experiments with three different bleach window sizes: 34.01 μm, 141.7 μm, and 242.91 μm. Using fluorescein-labeled dextrans of 40 kDa and 70 kDa molecular weight, we found that different bleach window sizes do not affect diffusion coefficient estimates determined by PyFRAP (Supplementary Fig. 6).

FRAP experiments can be executed over different spatial scales, from subcellular to tissue-level measurements. Our experiments were performed on spatial scales that are three orders of magnitude larger than the microscope's resolution limit. However, it is possible that FRAP experiments in very small samples with subcellular bleach areas may be affected by the imaging resolution, and future deconvolution-based approaches could be helpful to improve the measurement accuracy of PyFRAP in these cases.

Simulating tortuosity

The movement of molecules during FRAP experiments in biological samples is affected by obstacles such as cells, nuclei, or filopodia, and such tortuous molecule movements have been suggested to alter recovery rates and diffusion estimates[6].

To obtain a better understanding of how obstacles alter effective diffusion coefficients, we performed a simulation study in two- and three-dimensional geometries. We placed objects with a radius of $r_{\mathrm{Bead}} \approx$ 20 μm (similar to the dimensions of cells and beads used in the present study) in each geometry in three different ways: 1) Equally sized beads aligned as a regular grid (Supplementary Fig. 7a), 2) randomly placed within the domain with radii drawn from a cut-off normal distribution (Supplementary Fig. 7b,d), and 3) equally sized beads placed according to a hexagonal close-packing (Supplementary Fig. 7c). Beads were placed with different minimal gaps between them, ranging from 0.05 μm to 10 μm. For 2D simulations, the overall geometry was a circle with radius 300 μm. We chose a cylinder with equal radius and height of 100 μm or a cuboid with dimensions 600 $\mu m \times 600$ $\mu m \times 100$ μm for all 3D simulations experiments. The combination between various placement methods and gap sizes allowed us to vary the extracellular volume fraction (EVF) – i.e. the space available for the diffusing molecules – from 25% to 78%.

Confirming previous analyses[9–12], we found that the introduction of beads delayed molecule recovery in the bleached ROI, and the effect of tortuosity increased as the EVF decreased (Fig. 5b, Supplementary Fig. 7e,f, Supplementary Table 8). Moreover, the effect in two-dimensional experiments was more severe. For example, FRAP simulations with EVF = 36% reduced diffusion by 51% compared to only 40% for EVF = 38% in a three-dimensional simulation. Both observations are in line with theoretical predictions and previous results[9–12].

BSA does not affect fluorophore diffusivity

We found a stronger effect of bead-mediated tortuosity on 70 kDa fluorescein-labeled dextran molecules than on GFP *in vitro* (Fig. 5d,e). BSA was added to the aqueous solution with GFP to prevent the fluorescent protein from interacting with the plexiglass surface of the drilled hole in the *in vitro* experiments. To test whether BSA might also interact with the polyacrylamide beads and thus distort FRAP results, we repeated the experiments with 70 kDa fluorescein-labeled dextran both for pure diffusion with beads in addition to experiments with 70 kDa fluorescein-labeled dextran + BSA + beads. We found that BSA had no influence on the recovery rates, yielding equal results within standard error, i.e. 14.9 ± 2.1 µm^2/s for bead experiments and 15.1 ± 2.4 µm^2/s for experiments with additional BSA (Supplementary Fig. 9a).

Varying the experimental settings for Squint-GFP FRAP experiments does not consistently affect measured diffusion coefficients

For the FRAP experiments with Squint-GFP produced from injected mRNA, we acquired data sets varying the amount of injected mRNA, the frame rate and length of image acquisition, and the zoom factor of the microscope. Results were partitioned into three experimental groups, i.e. images recorded with 1) a frame rate of 1 frames/10 s for 3000 s with 30 pg of injected mRNA and a spatial resolution of 340.08 µm $\times$ 340.08 µm, 2) a frame rate of 1 frame/10 s for 3000 s with 200 pg of injected mRNA and a spatial resolution of 566.79 µm $\times$ 566.79 µm, and 3) a frame rate of 1 frame/s for 300 s with 200 pg of injected mRNA and a spatial resolution of 566.79 µm $\times$ 566.79 µm.

There were no clear trends between different acquisition methods (Supplementary Fig. 9b). However, acquiring images at a higher frame rate for a shorter period of time appeared to make experiments and thus apparent diffusion coefficients more noisy, possibly resulting from the slow transport process underlying Squint-GFP diffusion.

Supplementary Note 4: PyFRAP analysis speed

To evaluate PyFRAP's analysis speed, we tested several analysis settings on different operating systems and computers. We designed three test cases: 1) A two-dimensional circular geometry similar to those used for the benchmarking simulations described in Supplementary Note 2, 2) a three-dimensional frustum geometry identical to the ones used to analyse the *in vitro* FRAP experiments described in the present work, and 3) a three-dimensional geometry resembling a zebrafish embryo at dome stage similar to our analysis of the *in vivo* experiments. The test data sets had identical properties as the data described for the respective experiments. A summary of all relevant test parameters can be found in Supplementary Table 13. All cases were tested on the three common operating systems Mac OSX, Microsoft Windows, and Ubuntu Linux, and the time from analysing the image data to mesh generation, simulation, and model fitting was measured for each test case. The results of these tests are summarised in Supplementary Table 14.

Note that PyFRAP does not allow parallel processing and only uses a single core of a CPU.

Supplementary Table 1. Combinations of image correction and smoothing methods used to analyse FRAP experiments. See Supplementary Fig. 3 for the results of this analysis. Note that flattening and normalisation were never applied at the same time since this would have distorted the image data.

Combination	Normalisation	Flattening	Background subtraction	Gaussian filter	Median filter
1	Off	Off	Off	Off	Off
2	Off	Off	Off	Off	On
3	Off	Off	Off	On	Off
4	Off	Off	Off	On	On
5	Off	Off	On	Off	Off
6	Off	Off	On	Off	On
7	Off	Off	On	On	Off
8	Off	Off	On	On	On
9	Off	On	Off	Off	Off
10	Off	On	Off	Off	On
11	Off	On	Off	On	Off
12	Off	On	Off	On	On
13	Off	On	On	Off	Off
14	Off	On	On	Off	On
15	Off	On	On	On	Off
16	Off	On	On	On	On
17	On	Off	Off	Off	Off
18	On	Off	Off	Off	On
19	On	Off	Off	On	Off
20	On	Off	Off	On	On
21	On	Off	On	Off	Off
22	On	Off	On	Off	On
23	On	Off	On	On	Off
24	On	Off	On	On	On

Supplementary Table 2. Statistical tools available in PyFRAP.

Method	Purpose	Type	Publication
Student's t-test	Significance testing	Parametric	[13]
Welch's t-test	Significance testing	Parametric	[14]
Wilcoxon signed-rank test	Significance testing	Non-parametric	[15]
Mann-Whitney U test	Significance testing	Non-parametric	[16]
Shapiro-Wilk test	Normality testing	Parametric	[17]
Akaike Information Criterion (AIC)	Model comparison	Parametric	[18]

Supplementary Table 3. Benchmarking PyFRAP against an in-house software combination of MATLAB and COMSOL Multiphysics. Data was simulated with MATLAB and COMSOL Multiphysics[5–7], and then fitted with PyFRAP for each of the four available reaction-diffusion models.

MATLAB + COMSOL Multiphysics			*PyFRAP*			*R^2-value*		*AIC*
**D (μm^2/s)**	**Degradation (10^{-4}/s)**	**Production (10^{-4} [c]/s)**	**D (μm^2/s)**	**Degradation (10^{-4}/s)**	**Production (10^{-4} [c]/s)**	**Bleached window**	**Slice**	**Correct model prediction**
Pure diffusion								
1	0	0	1.0	0	0	0.998	0.972	yes
5	0	0	4.8	0	0	1.000	0.910	yes
10	0	0	9.5	0	0	1.000	0.932	yes
40	0	0	39.1	0	0	0.999	0.870	yes
110	0	0	109.4	0	0	0.999	0.984	yes
200	0	0	199.1	0	0	0.999	0.990	yes
Diffusion + degradation								
1	5.0	0	1.0	5.8	0	0.998	0.921	yes
5	5.0	0	4.9	5.5	0	1.000	0.959	yes
10	5.0	0	9.7	5.4	0	1.000	0.972	yes
40	5.0	0	39.0	5.0	0	0.999	0.950	yes
110	5.0	0	108.1	4.9	0	0.999	0.943	yes
200	5.0	0	198.0	5.0	0	0.999	0.982	yes
Diffusion + production								
1	0	5.0	1.0	0	4.4	0.999	0.950	yes
5	0	5.0	5.0	0	4.6	1.000	0.972	yes
10	0	5.0	9.8	0	4.7	1.000	0.978	yes
40	0	5.0	38.9	0	5.0	1.000	0.991	yes
110	0	5.0	108.3	0	5.1	1.000	0.998	yes
200	0	5.0	198.4	0	5.0	1.000	0.999	yes
Diffusion + production + degradation								
1	5.0	7.0	1.1	4.8	6.2	0.992	0.845	no
5	5.0	7.0	5.0	4.8	6.4	1.000	0.894	no
10	5.0	7.0	9.5	5.9	7.5	1.000	0.910	no
40	5.0	7.0	39.2	4.1	6.3	1.000	0.979	no
110	5.0	7.0	105.2	9.7	12.0	0.999	0.995	no
200	5.0	7.0	192.6	11.6	13.3	1.000	0.996	no

Supplementary Table 4. Selection of current FRAP analysis software packages.

Software	Fit type	Result type	Input data	Publication	Platform	Tested	Comments
easyFRAP	Analytical	Qualitive	CSV	[1]	Windows, Mac OSX	Yes	Requires MATLAB Runtime, only produces $\tau_{1/2}$
FrapCalc	Analytical	Qualitive	CSV	[2]	Windows, Mac OSX	Yes	Requires IgorPro
FRAP	Analytical	Qualitive	Image files	[19]	Cross-platform	No	Requires specialised MATLAB toolboxes
simFRAP	Simulation	Quantitive	Image files	[4]	Cross-platform	Yes	Fiji Plugin
virtualFRAP	Simulation	Quantitive	Image files	[3]	Windows	Yes	
FRAPToolbox	Simulation	Quantitive	Image files	[20]	Cross-platform	No	Unable to read non-OME formats
Tropical	Simulation	Quantitive	Image files	[21]	Windows, Linux	No	Software unavailable

Supplementary Table 5. Fluorescent samples used for *in vitro* experiments, and their calculated theoretical diffusion coefficients. Theoretical values were only computed if an estimate of the molecule's Stokes radius could be found (see Supplementary Note 3 for details).

Fluorophore	Molecular weight (kDa)	Concentration (µM)	Manufacturer	Stokes radius (nm)	Theoretical D ($\mu m^2/s$)
Fluorescein-dextran	3	1	Thermo Fisher	1.36	171
Fluorescein-dextran	4	1, 15, 100	Sigma-Aldrich	1.4	166
Fluorescein-dextran	10	1	Thermo Fisher	2.3	101
Fluorescein-dextran	40	1	Thermo Fisher	4.5	52
Fluorescein-dextran	70	1	Sigma-Aldrich	6.0	39
Fluorescein-dextran	70	1	Thermo Fisher	6.0	39
Fluorescein-dextran	150	1	Sigma-Aldrich	8.5	27
Fluorescein-dextran	500	1	Thermo Fisher	15.8	15
GFP	32.7	4	Biovision	n.a.	n.a.
Dendra2	27.5	0.5	Hoelzel Diagnostics	n.a	n.a.

Supplementary Table 6. Diffusion coefficients determined by *in vitro* experiments and PyFRAP analysis. Theoretical values were only computed if an estimate of the molecule's Stokes radius could be found. Mean D values determined by PyFRAP as well as literature values are given with standard deviation.

			PyFRAP		***Literature***		
**Dextran size (kDa)**	**Manufacturer**	**D (µm²/s) theoretical**	**D (µm²/s) experimental**	**n**	**D (µm²/s) experimental**	**Technique**	**Reference**
3	Thermo Fisher	171	170.3 ± 21.9	19	161 ± 22	FCS	[22]
4	Sigma-Aldrich	166	181.1 ± 31.6	44	135 ± 10	FRAP	[23]
10	Thermo Fisher	101	83.1 ± 8.0	12	122 ± 4	FCS	[22]
40	Thermo Fisher	52	45.3 ± 11.1	57	47 ± 2	FCS	[22]
70	Thermo Fisher	39	26.9 ± 4.9	35	37 ± 7	FCS	[22]
70	Sigma-Aldrich	39	49.2 ± 5.6	31	30 ± 2	FRAP	[24]
150	Sigma-Aldrich	27	46.4 ± 5.6	31	26 ± 2	FRAP	[24]
500	Thermo Fisher	15	25.7 ± 1.8	11	23.2 ± 1.1	FRAP	[25]

Supplementary Table 7. Literature values used for Fig. 5.

Molecule	MW (kDa)	Temperature during measurement (°C)	Manufacturer	D ($\mu m^2/s$)	Stdev ($\mu m^2/s$)	Technique	Reference
Fluorescein	0.33	22	Sigma-Aldrich	300	n.a.	FCS	[24]
Fluorescein	0.33	23	n.a.	270	n.a.	FRAP	[26]
Fluorescein	0.33	23	n.a.	260	n.a.	FRAP	[26]
Na2-Fluorescein	0.376	25	Fluka	380	35	FRAP	[27]
Oregon Green 488 carboxylic acid	0.41230	23	Thermo Fisher	336	11	FCS	[22]
Rhodamine B	0.47901	23	Fluka	420	20	FCS	[22]
Rhodamine B	0.47901	22.5	Sigma-Aldrich	420	30	FCS	[28]
Rhodamine 6 G	0.47901	22.5	Molecular Probes	400	30	FCS	[28]
Rhodamine 6 G	0.47901	23	Thermo Fisher	400	20	FCS	[22]
Tetramethyl-Rhodamine methyl ester	0.50093	23	Thermo Fisher	412	18	FCS	[22]
Oregon Green 488 carboxylic acid succinimidyl ester	0.50938	23	Thermo Fisher	308	10	FCS	[22]
Rhodamine green succinimidyl ester	0.621	20	Molecular Probes	233	3	FCS	[29]
Alexa488 alkyne	0.774	32	Life Technologies	288	8	FCS	[30]
Fluorescent dextran	3	23	Thermo Fisher	161	22	FCS	[22]
Alexa488-dextran	3	32	Life Technologies	160	5	FCS	[30]
FITC-dextran	3	22	Pharmacia	98	6	FRAP	[31]
FITC-dextran	3	n.a.	Pharmacia	98	6	FRAP	[32]
FITC-dextran	4	25	Sigma-Aldrich	149	n.a.	FRAP	[33]
FITC-dextran	4	25	Sigma-Aldrich	135	10	FRAP	[23]
FITC-dextran	4	32	Sigma-Aldrich	135	6	FCS	[30]
FITC-dextran	4	20	Sigma-Aldrich	96	2.4	FCS	[34]
FITC-dextran	4	22	Sigma-Aldrich	89	n.a.	FRAP	[35]
FITC-dextran	4	19	Sigma-Aldrich	155	23	FRAP	[36]
FITC-dextran	9.4	20	Sigma-Aldrich	75	3	FRAP	[37]
Fluorescent dextran	10	23	Thermo Fisher	122	4	FCS	[22]
Rhodamine green dextran	10	20	Molecular Probes	115	4	FCS	[29]
Alexa488-dextran	10	32	Life Technologies	82	1.4	FCS	[30]
FITC-dextran	10	22	Sigma-Aldrich	76	n.a.	FRAP	[31]
FITC-dextran	10	20	Sigma-Aldrich	68	1	FCS	[34]
FITC-dextran	11	n.a.	Sigma-Aldrich	76	2.5	FRAP	[32]
FITC-dextran	11	22	Sigma-Aldrich	76	3	FRAP	[31]
FITC-dextran	12	25	Sigma-Aldrich	97	n.a.	FRAP	[33]
FITC-Insulin	12	25	Sigma-Aldrich	147	13	FRAP	[33]
FITC-dextran	17	22	Sigma-Aldrich	65	n.a.	FRAP	[31]
FITC-dextran	17.2	20	Sigma-Aldrich	64	2	FRAP	[37]
FITC-dextran	18	22	Sigma-Aldrich	65	7	FRAP	[31]
FITC-dextran	18	n.a.	Sigma-Aldrich	65	6.5	FRAP	[32]
FITC-dextran	20	22	Sigma-Aldrich	78	n.a.	FCS	[24]
FITC-dextran	20	22	Sigma-Aldrich	64	2	FRAP	[24]
FITC-dextran	20	29	Sigma-Aldrich	70	8	FRAP	[36]
FITC-dextran	20	22	Sigma-Aldrich	63	4	FRAP	[25]
FITC-dextran	21	25	Sigma-Aldrich	71	n.a.	FRAP	[33]
GFP	26.9	25	custom-made	87	n.a.	FCS	[38]
GFP	26.9	n.a.	custom-made	87	n.a.	FRAP	[39]
GFP	26.9	22	Clontech	82	n.a.	FCS	[24]

FITC-dextran	35.6	20	Sigma-Aldrich	44	5	FRAP	[37]
FITC-dextran	38	25	Sigma-Aldrich	62	n.a.	FRAP	[33]
Fluorescent dextran	40	23	Thermo Fisher	47	2	FCS	[22]
FITC-dextran	40	22	Sigma-Aldrich	45	n.a.	FCS	[40]
FITC-dextran	40	22	Sigma-Aldrich	45	n.a.	FCS	[24]
FITC-dextran	40	32	Sigma-Aldrich	45	1.1	FCS	[30]
FITC-dextran	40	22	Sigma-Aldrich	44	5	FRAP	[24]
FITC-dextran	40	22	Sigma-Aldrich	52	2	FRAP	[25]
FITC-dextran	41	22	Sigma-Aldrich	46	5	FRAP	[31]
FITC-dextran	41	n.a.	Sigma-Aldrich	46	4.6	FRAP	[32]
FITC-dextran	42	20	Sigma-Aldrich	39	0.4	FCS	[34]
FITC-dextran	51	25	Sigma-Aldrich	54	n.a.	FRAP	[33]
FITC-dextran	62	n.a.	Sigma-Aldrich	39	2.6	FRAP	[32]
FITC-dextran	62	22	Sigma-Aldrich	39	3	FRAP	[31]
FITC-BSA	67	25	n.a.	58	5	FRAP	[33]
FITC-dextran	70	22	Sigma-Aldrich	38	n.a.	FCS	[24]
FITC-dextran	70	22	Sigma-Aldrich	38	n.a.	FCS	[40]
Fluorescent dextran	70	23	Thermo Fisher	37	7	FCS	[22]
FITC-dextran	70	n.a.	Fluka	33	2.1	FCS	[41]
FITC-dextran	70	22	Sigma-Aldrich	30	2	FRAP	[24]
FITC-dextran	70	25	Thermo Fisher	30	3.1	FRAP	[27]
FITC-dextran	70	23	n.a.	23	n.a.	FRAP	[26]
FITC-dextran	70	22	Sigma-Aldrich	44	1	FRAP	[25]
FITC-dextran	71	25	Sigma-Aldrich	44	2	FRAP	[23]
FITC-dextran	71.2	20	Sigma-Aldrich	30	2	FRAP	[37]
FITC-dextran	77	20	Sigma-Aldrich	35	0.6	FCS	[34]
FITC-dextran	148	20	Sigma-Aldrich	25	3.1	FCS	[34]
FITC-dextran	148	20	Sigma-Aldrich	18	1	FRAP	[37]
FITC-dextran	150	22	Sigma-Aldrich	26	2	FRAP	[24]
FITC-dextran	150	22	Sigma-Aldrich	24	n.a.	FCS	[40]
FITC-dextran	150	22	Sigma-Aldrich	24	n.a.	FCS	[24]
FITC-dextran	150	20	Sigma-Aldrich	14	n.a.	FRAP	[42]
FITC-dextran	157	n.a.	Sigma-Aldrich	24	1.3	FRAP	[32]
FITC-dextran	157	22	Sigma-Aldrich	24	1	FRAP	[31]
FITC-dextran	167	25	Sigma-Aldrich	38	n.a.	FRAP	[33]
FITC-dextran	167	n.a.	Sigma-Aldrich	18.8	0.2	FRAP	[43]
FITC-dextran	260	25	Sigma-Aldrich	30	n.a.	FRAP	[33]
FITC-dextran	282	20	Sigma-Aldrich	16.6	0.8	FCS	[34]
FITC-dextran	464	20	Sigma-Aldrich	14	0.6	FCS	[34]
FITC-dextran	464	n.a.	Sigma-Aldrich	11	0.5	FRAP	[43]
FITC-dextran	500	22	Sigma-Aldrich	23	1	FRAP	[25]
FITC-dextran	580	25	Sigma-Aldrich	22	n.a.	FRAP	[33]
FITC-dextran	2000	25	Sigma-Aldrich	10	1	FRAP	[23]
Fluorescent dextran	2000	23	Thermo Fisher	6	1	FCS	[22]
FITC-dextran	2000	n.a.	Sigma-Aldrich	6.4	0.09	FRAP	[43]
FITC-dextran	2101	25	Sigma-Aldrich	14	n.a.	FRAP	[33]

Supplementary Table 8. Summary of tortuosity simulations.

Dimension	Geometry	Packing	Extracelluar volume fraction (EVF) (%)	Diffusion hindrance factor θ
2D	Circle	Regular	74	0.74
2D	Circle	Regular	59	0.61
2D	Circle	Random	56	0.57
2D	Circle	Random	36	0.49
2D	Circle	Ideal	25	0.44
3D	Cylinder	Regular	71	0.86
3D	Cylinder	Random	78	0.92
3D	Cylinder	Random	58	0.88
3D	Cylinder	Ideal	78	0.92
3D	Cylinder	Ideal	71	0.874
3D	Cylinder	Ideal	61	0.871
3D	Cylinder	Ideal	60	0.870
3D	Cylinder	Ideal	42	0.75
3D	Cuboid	Ideal	38	0.60

Supplementary Table 9. Diffusion coefficients determined by *in vitro* experiments and PyFRAP analysis in the presence of polyacrylamide beads. Mean diffusion values are given with standard error.

**Dextran size (kDa)**	**Manufacturer**	**Condition**	**D (μm^2/s)**	**n**
70	Thermo Fisher	Free	24.1 ± 0.4	13
70	Thermo Fisher	Beads	14.9 ± 0.5	17

Supplementary Table 10. Diffusion coefficients determined by *in vitro* and *in vivo* experiments and PyFRAP analysis with GFP and GFP fusion proteins. Mean diffusion values are given with standard error.

**Molecule**	**Manufacturer**	**Source**	**Condition**	**Context**	D (**µm^2/s**) **PyFRAP**	**n**
Recombinant GFP	Biovision	Protein	Free	*In vitro*	96.1 ± 2.2	23
Recombinant GFP	Biovision	Protein	Beads	*In vitro*	79.2 ± 4.1	18
Recombinant GFP	Biovision	Injected protein	Extracelluar matrix	*In vivo*	37.6 ± 3.7	15
Secreted GFP	In-house	Injected mRNA	Extracelluar matrix + production	*In vivo*	35.3 ± 4.8	17
Squint-GFP	In-house	Injected mRNA	Extracelluar matrix + production + binding	*In vivo*	1.7 ± 0.25	27

Supplementary Table 11. Parameters used for the simulation of FRAP experiments.

Variable	Definition	Default value
	Simulation	
D	Diffusion coefficient	$D = 50$ pixels^2/s
	Time stepping	
$t_{\text{sim,start}}$	Simulation start time	0 s
$t_{\text{sim,end}}$	Simulation end time	1680 s
n_{sim}	Number of time steps	4000
t_{scale}	Time-stepping scheme	Logarithmic
	Geometry	
r_{upper}	Upper radius of frustum	317.65 pixels
r_{lower}	Lower radius of frustum	224.25 pixels
h	Height of frustum	90.33 pixels
	Meshing	
v	Mesh element size	25 pixels^3
v_{BL}	Boundary layer element size	15 pixels^3
v_{slice}	Slice refinement element size	15 pixels^3
w_{BL}	Boundary layer thickness	30 pixels
	Solver	
ϵ	Solver tolerance	10^{-10}
N_{iter}	Solver iterations	1000

Supplementary Table 12. Fitting and model parameters, initial guesses, and bounded ranges. Note that we tried different initial guesses for the diffusion coefficient D, which prevented the minimisation algorithm from stopping at a local minimum. We then then took the fit that yielded the global minimum SSD.

Initial guesses		
**Parameter**	**Initial guess**	**Allowed range**
D (pixels^2/s)	1 - 200	0.01 - 400
k_1 (1/s)	0	0 - 100
k_2 ($[c]$/s)	0	0 - 100
E_{bleached}	1	0.1 - 3
E_{slice}	1	0.1 - 3
Fitting convergence		
**Parameter**	**Definition**	**Default value**
N_{max}	Maximum number of function calls	1000
δ	Tolerance of termination	10^{-10}

Supplementary Table 13. Test data and settings to measure PyFRAP analysis speed.

	2D	Frustum	Dome
**Geometry**	2D circle	3D frustum	3D zebrafish dome
**Number of images**	301	301	301
**Number of mesh cells**	7000	20000	35000
**Number of time steps**	1000	3000	3000
**Illumination correction**	No	Yes	Yes
**Median filter application**	No	Yes	Yes

Supplementary Table 14. PyFRAP analysis speed.

Operating system	Version	Processor	Memory	2D test (s)	Frustum test (s)	Dome test (s)
Ubuntu	14.04 LTS	Intel Core i7-3520M 2.90 GHz	8 GB	97	378	489
Ubuntu	16.04 LTS	Intel Core i5-4210 2.60 GHz	8 GB	125	521	743
Ubuntu	16.04 LTS	Intel Xeon E3-1275 3.60 GHz	64 GB	73	347	437
Mac OS X	10.13.3	Intel Core i7-4790K 4.00 GHz	32 GB	79	282	386
Windows	8.1	Intel Core i7-5600U 2.60 Ghz	8 GB	91	373	567

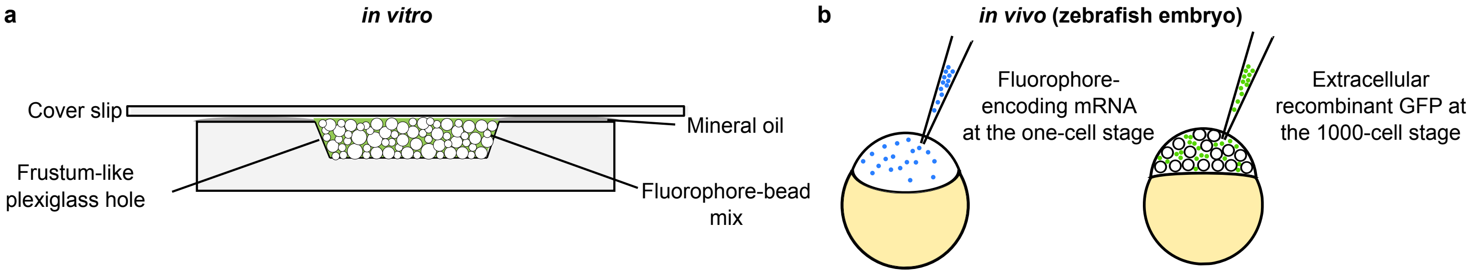

Supplementary Figure 1 | Sample preparation for *in vitro* and *in vivo* FRAP experiments. (a) *In vitro* experiments. Fluorophore solution was pipetted into a frustum-like plexiglass hole. The hole was then sealed with mineral oil and covered with a cover slip. The sample was flipped and placed under an inverted confocal microscope. **(b)** *In vivo* experiments in zebrafish embryos. mRNA encoding a fluorophore was injected into embryos at the one-cell stage, or recombinant GFP was injected into the extracellular space of embryos at the 1000-cell stage.

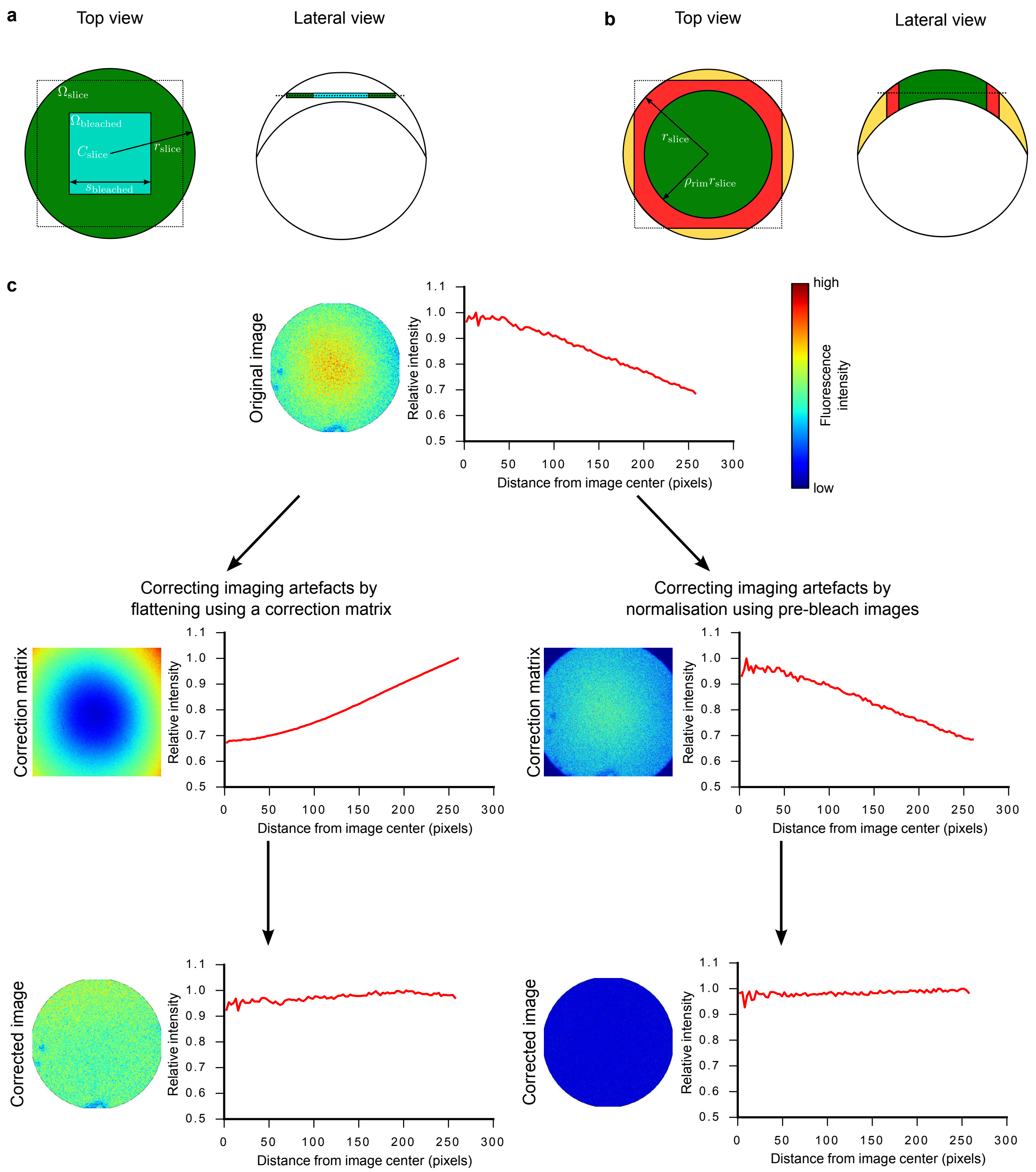

Supplementary Figure 2 | Image analysis in PyFRAP. (a) Basic regions of interest (ROIs) of FRAP analysis: The cyan square indicates the bleached region of the FRAP experiment inside the complete circular geometry within the imaging slice. The dashed lines indicate the location of the acquired image data. **(b)** Rim concentration calculation: Hypothetical data (orange) outside the acquired image (dashed line) is extrapolated through the average concentration in a slim rim of the visible fraction in the imaging slice (red). **(c)** Image manipulation techniques used to correct uneven illumination: Correction was either performed by multiplying the data with a correction matrix (flattening), or by dividing the data through an average pre-bleach image (normalisation). The original image shows a pre-bleach measurement of a uniformly distributed fluorophore. Deviations from the theoretical flat intensity profile are due to imaging artefacts.

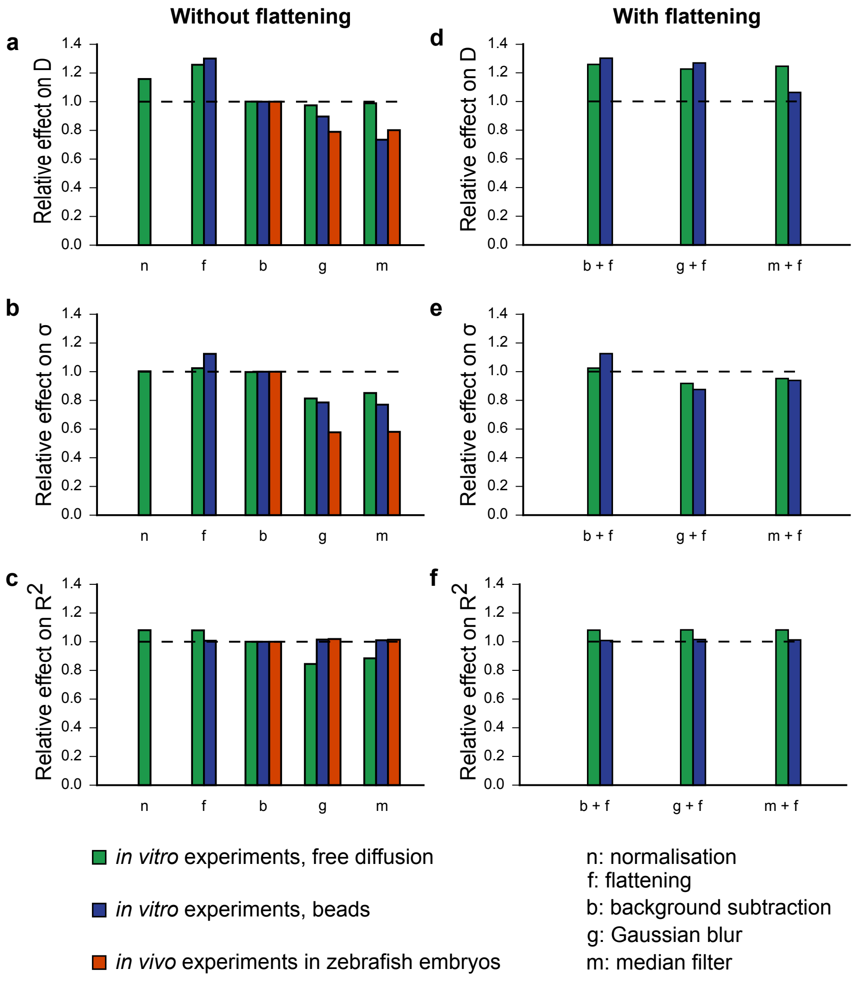

Supplementary Figure 3 | Analysis subset of image correction and smoothing techniques. Data sets were grouped by condition (*in vitro* experiments with free diffusion (green), *in vitro* experiments with beads (blue), and *in vivo* experiments in zebrafish embryos (orange)). Bar plots show the effect of each manipulation (n: normalisation, f: flattening, b: background subtraction, g: Gaussian blur, m: median filter) compared to analyses in which no manipulation was applied. Values above or below the dashed line indicate that the manipulation had an effect. **(a,b,c)** Effect on mean diffusion coefficient D, standard deviation σ, and R^2-value if only one of the five image manipulation techniques was applied, respectively. **(e,d,f)** Effect if flattening and one of the three remaining manipulation techniques was applied. *In vivo* experiments with zebrafish embryos were excluded for this analysis (see Supplementary Note 1 for details).

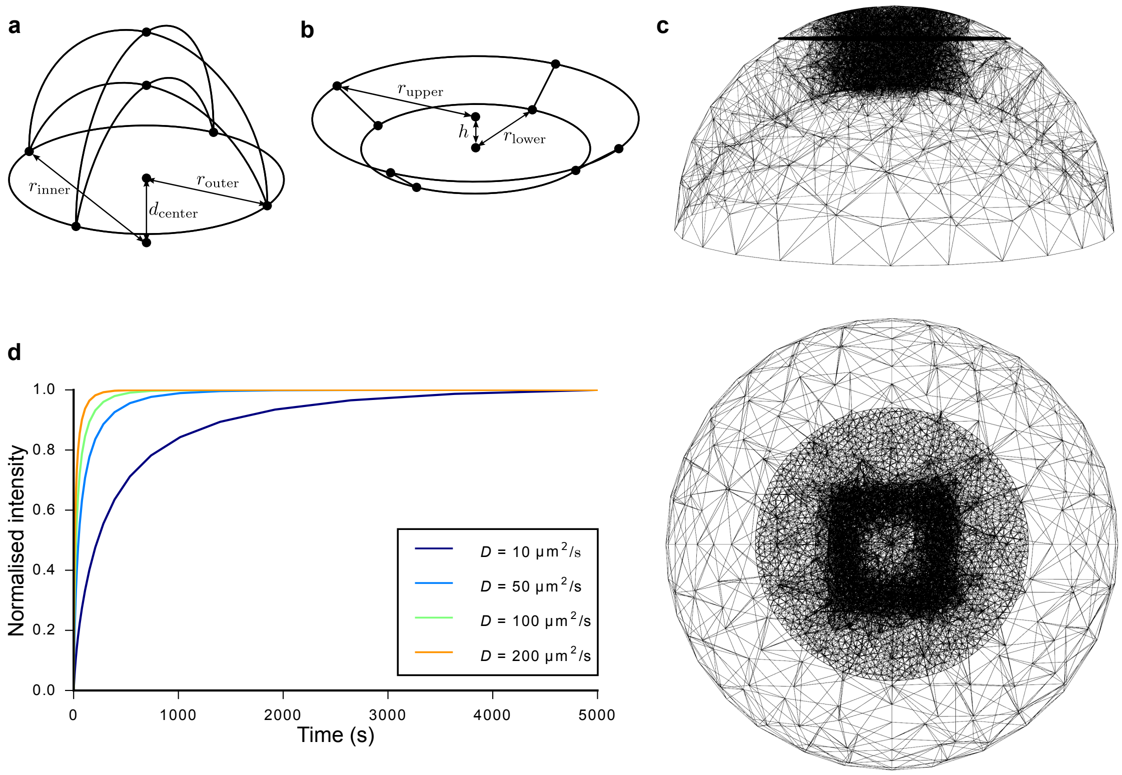

Supplementary Figure 4 | Simulation details for PyFRAP analysis. (a) The zebrafish dome geometry used to analyse *in vivo* experiments is described by the distance between the centers (d_{center}) and the radii (r_{inner}, r_{outer}) of two hemispheres. **(b)** The frustum geometry used to analyse *in vitro* experiments is described by the upper (r_{upper}) and lower (r_{lower}) radius and its height h. **(c)** Lateral and top views of tetrahedral meshes in the zebrafish dome geometry with a boundary layer mesh around the bleached area and a refined mesh in the imaging slice. **(d)** Scaling solution of a simulated FRAP recovery curve for different diffusion coefficients.

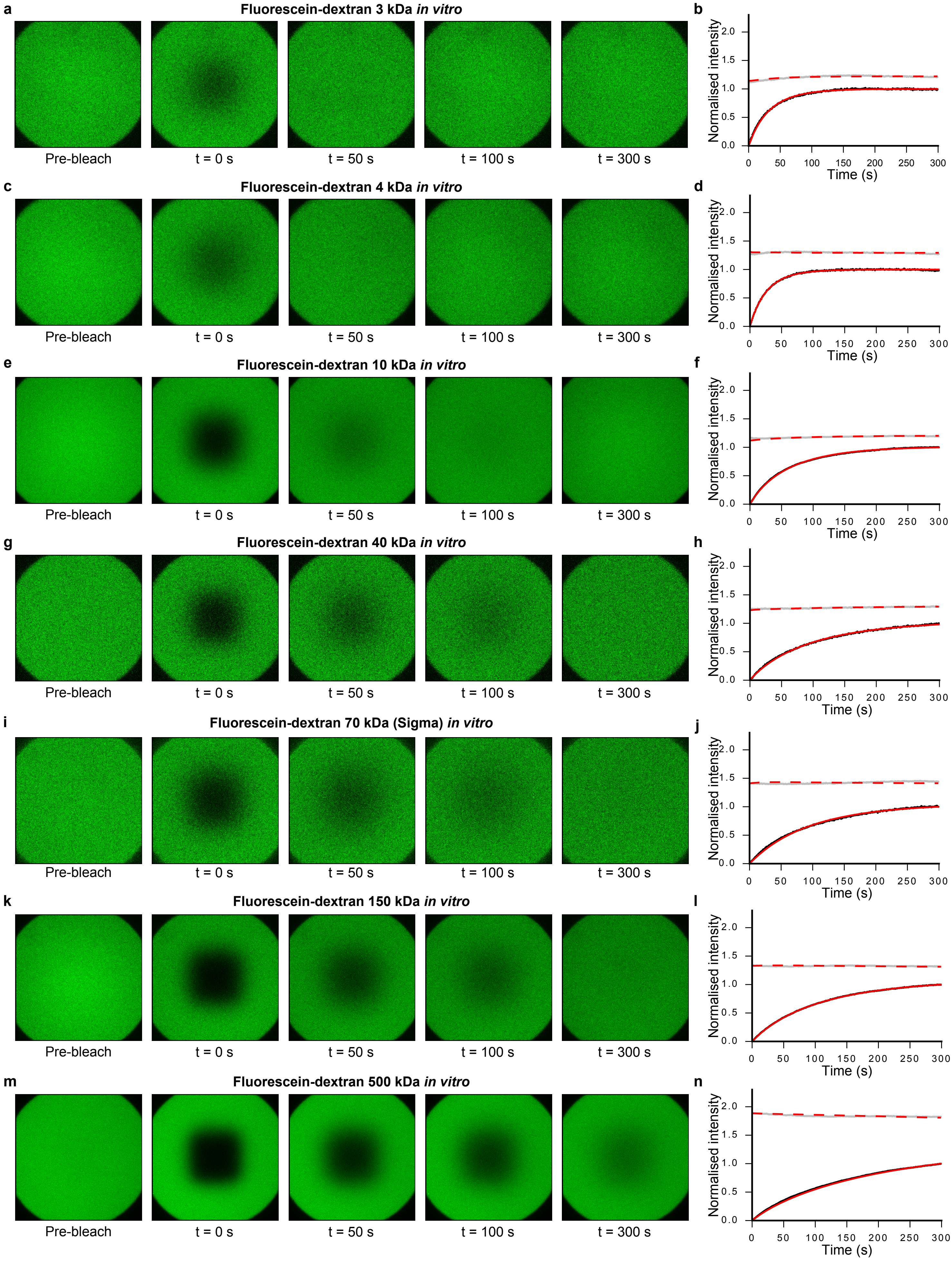

Supplementary Figure 5 | Examples of *in vitro* experiments and the resulting fits to measure free diffusion. (a,c,e,g,i,k,m) *In vitro* FRAP experiments with FITC-dextrans ranging from 3 kDa to 500 kDa. Maximum image intensities are the average pre-conversion intensities to facilitate comparison across data sets. **(b,d,f,h,j,l,n)** Black and grey dots represent data points of bleached and slice ROI, respectively. Red solid and dashed lines show the respective fits. Recovery curves were normalised between 0 (intensity in the bleached ROI at the first post-bleach time point) and 1 (intensity in the bleached ROI at the last post-bleach time point) to facilitate comparison across data sets.

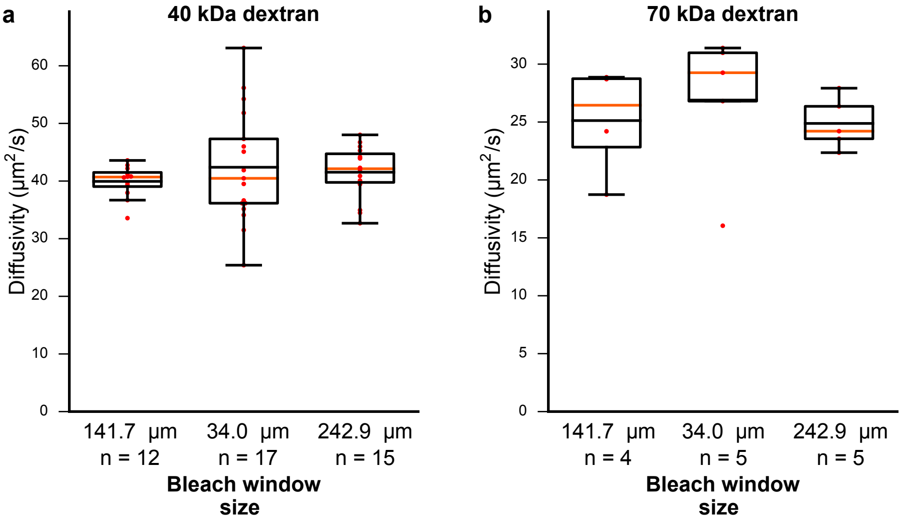

Supplementary Figure 6 | Different bleach window sizes do not affect diffusion coefficient estimates. (a) Results of control experiments with fluorescent dextran (40 kDa) for differently sized bleach windows. **(b)** Results of control experiments with fluorescent dextran (70 kDa) for differently sized bleach windows. Box plots in (a) and (b) show median (orange line), mean (black line), 25% quantiles (box), and all included data points (red markers). Whiskers extend to the smallest data point within the 1.5 interquartile range of the lower quartile, and to the largest data point within the 1.5 interquartile range of the upper quartile.

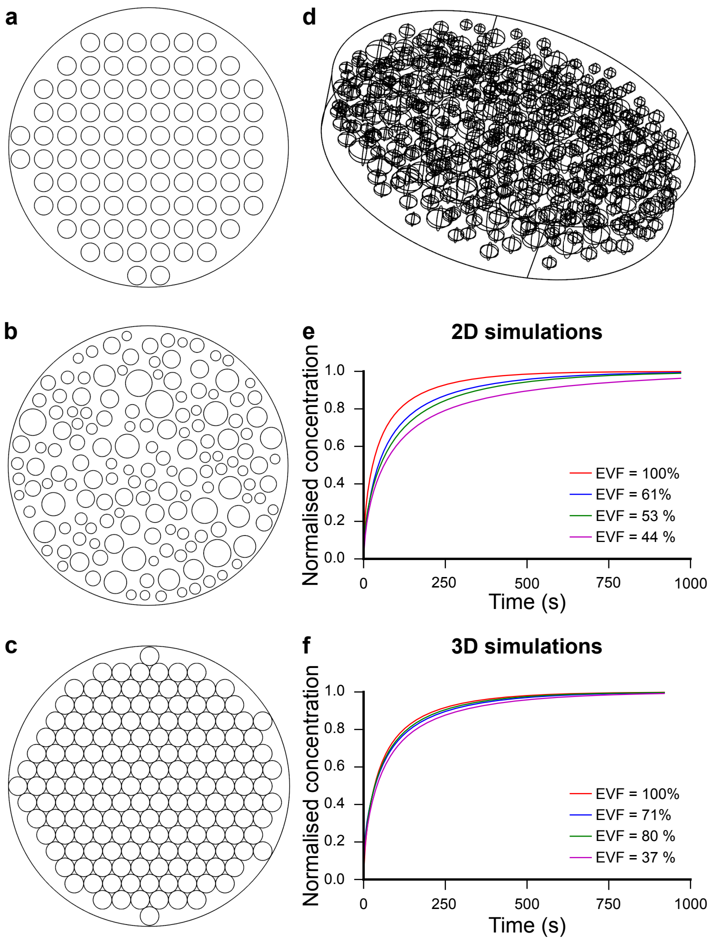

Supplementary Figure 7 | Simulations of tortuous environments in bead experiments. (a,b,c) Regularly (EVF = 59%), randomly (EVF = 56%), and ideally (EVF = 25%) placed beads in a two-dimensional circular domain. **(d)** Randomly (EVF = 78%) placed beads in a three-dimensional cylindrical domain. **(e,f)** Comparison between recovery curves in 2D and 3D bead simulations. Red lines indicate simulations without beads, blue lines indicate simulations with regularly placed beads, green lines indicate simulations with randomly placed beads, and magenta lines indicate simulations with ideally placed beads.

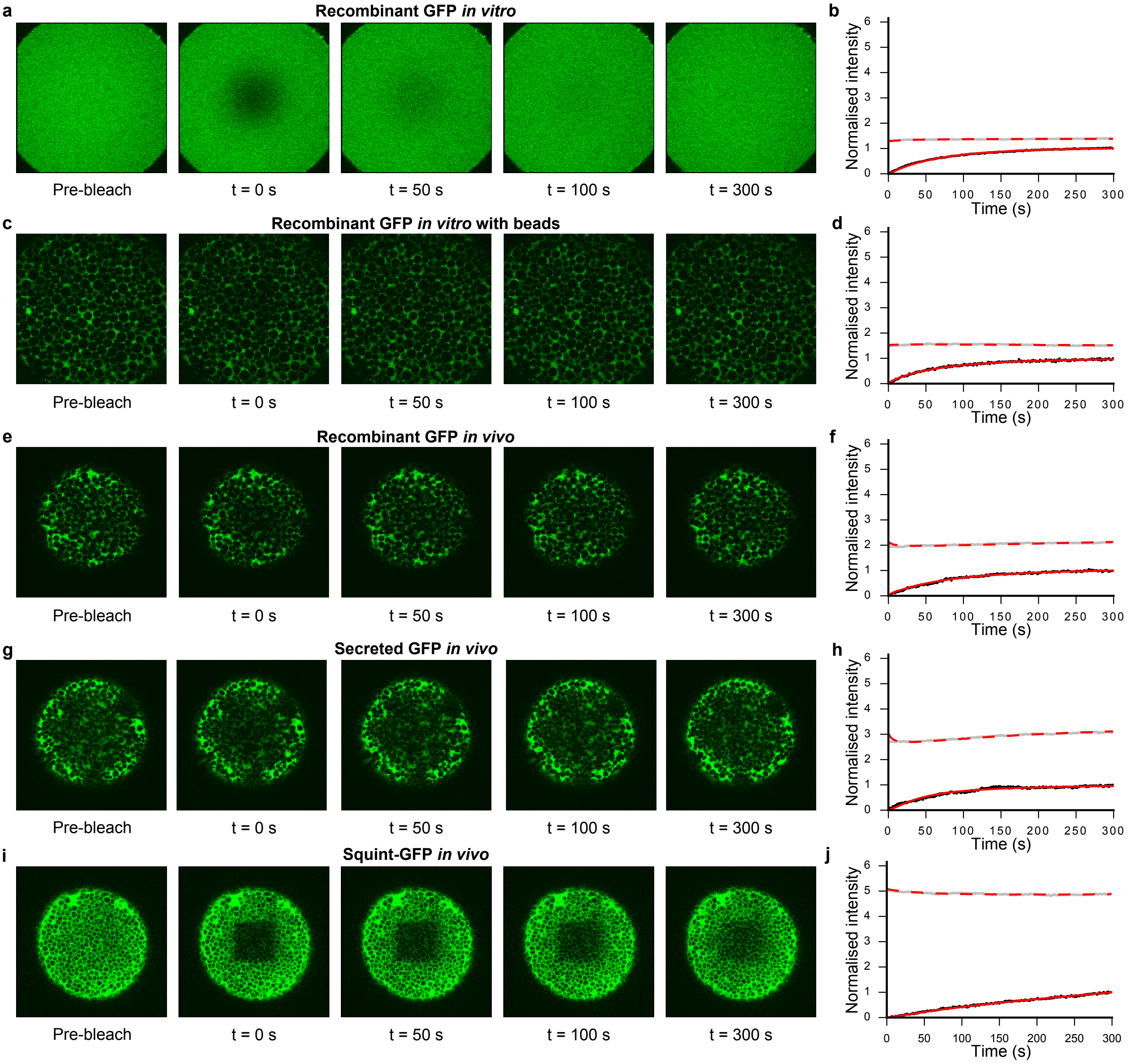

Supplementary Figure 8 | Examples of *in vitro* and *in vivo* experiments and the resulting fits. (a,b) *In vitro* FRAP experiment with recombinant GFP. **(c,d)** *In vitro* FRAP experiment with recombinant GFP mixed with polyacrylamide beads. **(e,f,g,h,i,j)** *In vivo* FRAP experiment in zebrafish embryos with recombinant GFP, secreted GFP, and Squint-GFP, respectively. **(b,d,f,h,j)** Black and grey dots represent data points of bleached and slice ROI, respectively. Red solid and dashed lines show the respective fits. Recovery curves were normalised between 0 (intensity in the bleached ROI at the first post-bleach time point) and 1 (intensity in the bleached ROI at the last post-bleach time point) to facilitate comparison across data sets.

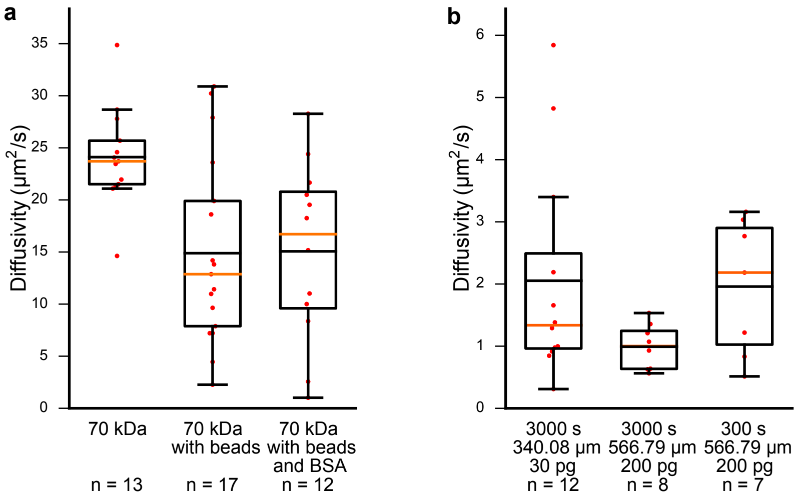

Supplementary Figure 9 | Results of control experiments for *in vitro* and *in vivo* FRAP experiments. (a) Results of control experiments with fluorescent dextran (70 kDa), and beads with or without BSA. BSA does not influence the diffusion of the fluorescent dextran. **(b)** Results of control experiments for different amounts (30 - 200 pg) of injected *Squint-GFP* mRNA, varying length of experiments (300 - 3000 s) and magnification (image size: 340.08 - 566.79 µm). Different imaging settings do not affect the measured diffusion coefficient of Squint-GFP. Box plots in (a) and (b) show median (orange line), mean (black line), 25% quantiles (box), and all included data points (red markers). Whiskers extend to the smallest data point within the 1.5 interquartile range of the lower quartile, and to the largest data point within the 1.5 interquartile range of the upper quartile.

Supplementary References

1. Rapsomaniki, M. A. *et al.* EasyFRAP: An interactive, easy-to-use tool for qualitative and quantitative analysis of FRAP data. *Bioinformatics* **28,** 1800–1801 (2012).
2. Miura, K. *FrapCalc* (accessed: 2016-12-12). `http://wiki.cmci.info/downloads/frap_analysis` (2016).
3. Schaff, J. C., Cowan, A. E., Loew, L. M. & Moraru, I. I. Virtual FRAP - an experiment-oriented simulation tool. *Biophysical Journal* **96,** 30a (2009).
4. Blumenthal, D., Goldstien, L., Edidin, M. & Gheber, L. A. Universal approach to FRAP analysis of arbitrary bleaching patterns. *Scientific Reports* **5,** 11655 (2015).
5. Müller, P. *et al.* Differential diffusivity of Nodal and Lefty underlies a reaction-diffusion patterning system. *Science* **336,** 721–724 (2012).
6. Müller, P., Rogers, K. W., Yu, S. R., Brand, M. & Schier, A. F. Morphogen transport. *Development* **140,** 1621–1638 (2013).
7. Pomreinke, A. P. *et al.* Dynamics of BMP signaling and distribution during zebrafish dorsal-ventral patterning. *Elife* **6,** e25861 (2017).
8. Sprague, B. L., Pego, R. L., Stavreva, D. A. & McNally, J. G. Analysis of binding reactions by Fluorescence Recovery After Photobleaching. *Biophysical Journal* **86,** 3473–3495 (2004).
9. Hrabe, J., Hrabetová, S. & Segeth, K. A model of effective diffusion and tortuosity in the extracellular space of the brain. *Biophysical Journal* **87,** 1606–1617 (2004).
10. Tao, L. & Nicholson, C. Maximum geometrical hindrance to diffusion in brain extracellular space surrounding uniformly spaced convex cells. *Journal of Theoretical Biology* **229,** 59–68 (2004).
11. Novak, I. L., Kraikivski, P. & Slepchenko, B. M. Diffusion in cytoplasm: Effects of excluded volume due to internal membranes and cytoskeletal structures. *Biophysical Journal* **97,** 758–767 (2009).
12. Donovan, P., Chehreghanianzabi, Y., Rathinam, M. & Zustiak, S. P. Homogenization theory for the prediction of obstructed solute diffusivity in macromolecular solutions. *PLoS ONE* **11,** e0146093 (2016).
13. Student. The probable error of a mean. *Biometrika* **6,** 1–25 (1908).
14. Welch, B. L. The generalisation of Student's problems when several different population variances are involved. *Biometrika* **34,** 28–35 (1947).
15. Wilcoxon, F. Individual comparisons by ranking methods. *Biometrics Bulletin* **1,** 80–83 (1945).
16. Mann, H. B. & Whitney, D. R. On a test of whether one of two random variables is stochastically larger than the other. *The Annals of Mathematical Statistics* **18,** 50–60 (1947).
17. Shapiro, S. S. & Wilk, M. B. An analysis of variance test for normality (complete samples). *Biometrika* **52,** 591–611 (1965).
18. Akaike, H. A new look at the statistical model identification. *IEEE Transactions on Automatic Control* **19,** 716–723 (1974).
19. Aaron, J. *FRAP* (accessed: 2016-12-12). `https://de.mathworks.com/matlabcentral/fileexchange/47327-frap-zip` (2016).
20. Kraft, L. J., Dowler, J. & Kenworthy, A. K. *Frap-Toolbox: Software for the analysis of Fluorescence Recovery After Photobleaching* (accessed: 2016-12-12). `http://www.fraptoolbox.com` (2014).

21. Ulrich, M. *et al.* Tropical-parameter estimation and simulation of reaction-diffusion models based on spatio-temporal microscopy images. *Bioinformatics* **22,** 2709–2710 (2006).

22. Zhang, Z., Nadezhina, E. & Wilkinson, K. J. Quantifying diffusion in a biofilm of Streptococcus mutans. *Antimicrobial Agents and Chemotherapy* **3,** 1075–1081 (2011).

23. Pluen, A., Netti, P. A., Jain, R. K. & Berk, D. A. Diffusion of macromolecules in agarose gels: Comparison of linear and globular configurations. *Biophysical Journal* **1,** 542–552 (1999).

24. Guiot, E. *et al.* Molecular dynamics of biological probes by Fluorescence Correlation Microscopy with two-photon excitation. *Journal of Fluorescence* **4,** 413–419 (2000).

25. Braga, J., Desterro, J. M. & Carmo-Fonseca, M. Intracellular macromolecular mobility measured by Fluorescence Recovery After Photobleaching with confocal laser scanning microscopes. *Molecular Biology of the Cell* **15,** 4749–4760 (2004).

26. Periasamy, N. & Verkman, A. Analysis of fluorophore diffusion by continuous distributions of diffusion doefficients: application to photobleaching measurements of multicomponent and anomalous diffusion. *Biophysical Journal* **1,** 557–567 (1998).

27. Schuster, E., Hermansson, A. M., Öhgren, C., Rudemo, M. & Lorén, N. Interactions and diffusion in fine-stranded β-lactoglobulin gels determined via FRAP and binding. *Biophysical Journal* **1,** 253–262 (2014).

28. Gendron, P. O., Avaltroni, F. & Wilkinson, K. J. Diffusion coefficients of several rhodamine derivatives as determined by pulsed field gradient-nuclear magnetic resonance and Fluorescence Correlation Spectroscopy. *Journal of Fluorescence* **6,** 1093–1101 (2008).

29. Visser, N. V., Hink, M. A., Hoek, A. V. & Visser, A. J. Comparison between Fluorescence Correlation Spectroscopy and time-resolved fluorescence anisotropy as illustrated with a fluorescent dextran conjugate. *Journal of Fluorescence* **3,** 251–255 (1999).

30. Kihara, T., Ito, J. & Miyake, J. Measurement of biomolecular diffusion in extracellular matrix condensed by fibroblasts using Fluorescence Correlation Spectroscopy. *PLoS ONE* **11** (2013).

31. Peters, R. Nucleo-cytoplasmic flux and intracellular mobility in single hepatocytes measured by fluorescence microphotolysis. *The EMBO Journal* **8,** 1831–6 (1984).

32. Lang, I., Scholz, M. & Peters, R. Molecular mobility and nucleocytoplasmic flux in hepatoma cells. *Journal of Cell Biology* **4,** 1183–1190 (1986).

33. Gribbon, P. & Hardingham, T. E. Macromolecular diffusion of biological polymers measured by confocal Fluorescence Recovery After Photobleaching. *Biophysical Journal* **2,** 1032–1039 (1998).

34. Gorisch, S. M. Histone acetylation increases chromatin accessibility. *Journal of Cell Science* **24,** 5825–5834 (2005).

35. Keminer, O. & Peters, R. Permeability of single nuclear pores. *Biophysical Journal* **1,** 217–228 (1999).

36. Floury, J., Madec, M. N., Waharte, F., Jeanson, S. & Lortal, S. First assessment of diffusion coefficients in model cheese by Fluorescence Recovery After Photobleaching (FRAP). *Food Chemistry* **2,** 551–556 (2012).

37. Arrio-Dupont, M., Cribier, S., Foucault, G., Devaux, P. & D'Albis, A. Diffusion of fluorescently labeled macromolecules in cultured muscle cells. *Biophysical Journal* **5,** 2327–2332 (1996).

38. Terry, B., Matthews, E. & Haseloff, J. Molecular characterization of recombinant green fluorescent protein by Fluorescence Correlation Microscopy. *Biochemical and Biophysical Research Communications* **1,** 21–27 (1995).

39. Swaminathan, R., Hoang, C. & Verkman, A. Photobleaching recovery and anisotropy decay of green fluorescent protein GFP-S65T in solution and cells: cytoplasmic viscosity probed by green fluorescent protein translational and rotational diffusion. *Biophysical Journal* **4,** 1900–1907 (1997).

40. Gulot, E. *et al.* Heterogeneity of diffusion inside microbial biofilms determined by Fluorescence Correlation Spectroscopy under two-photon excitation. *Photochemistry and Photobiology* **6,** 570–8 (2002).

41. Müller, K. P. *et al.* Multiscale analysis of dynamics and interactions of heterochromatin protein 1 by fluorescence fluctuation microscopy. *Biophysical Journal* **11,** 2876–2885 (2009).

42. Waharte, F., Steenkeste, K., Briandet, R. & Fontaine-Aupart, M. P. Diffusion measurements inside biofilms by image-based Fluorescence Recovery After Photobleaching (FRAP) analysis with a commercial confocal laser scanning microscope. *Applied and Environmental Microbiology* **17,** 5860–5869 (2010).

43. Braeckmans, K., Peeters, L., Sanders, N. N., De Smedt, S. C. & Demeester, J. Three-dimensional Fluorescence Recovery After Photobleaching with the confocal scanning laser microscope. *Biophysical Journal* **4,** 2240–2252 (2003).
